# Metabolomics Unveils Disrupted Pathways in Parkinson’s Disease: Toward Biomarker-Based Diagnosis

**DOI:** 10.1021/acschemneuro.4c00355

**Published:** 2024-08-23

**Authors:** Wanderleya T. Santos, Albert Katchborian-Neto, Gabriel S. Viana, Miller S. Ferreira, Luiza C. Martins, Thiago C. Vale, Michael Murgu, Danielle F. Dias, Marisi G. Soares, Daniela A. Chagas-Paula, Ana C. C. Paula

**Affiliations:** † Department of Pharmaceutical Sciences, Federal University of Juiz de Fora, Juiz de Fora 36036-900, Brazil; ‡ Chemistry Institute, Federal University of Alfenas, Alfenas 37130-001, Brazil; § Faculty of Medicine, Federal University of Juiz de Fora, Juiz de Fora 36036-900, Brazil; ∥ Waters Corporation, Barueri 06455-020, Brazil

**Keywords:** metabolomics, Parkinson’s disease, biomarkers, caffeine metabolism, multivariate analysis, machine learning

## Abstract

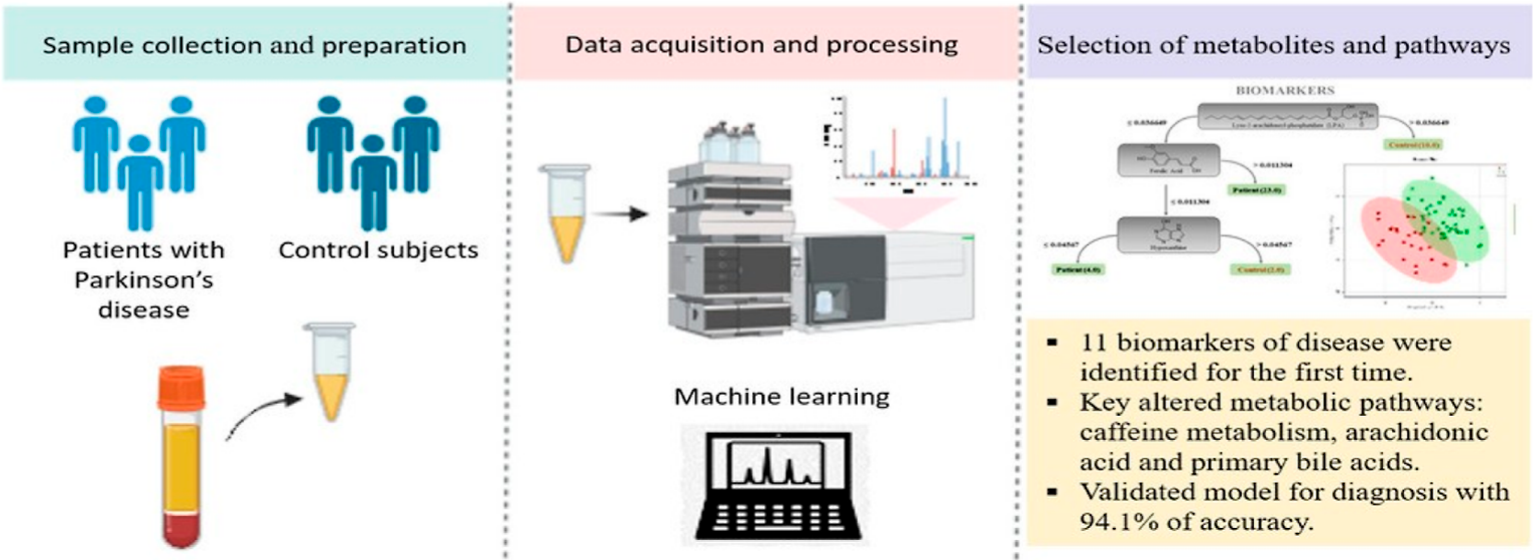

Parkinson’s disease (PD) is a neurodegenerative disorder characterized by diverse symptoms, where accurate diagnosis remains challenging. Traditional clinical observation methods often result in misdiagnosis, highlighting the need for biomarker-based diagnostic approaches. This study utilizes ultraperformance liquid chromatography coupled to an electrospray ionization source and quadrupole time-of-flight untargeted metabolomics combined with biochemometrics to identify novel serum biomarkers for PD. Analyzing a Brazilian cohort of serum samples from 39 PD patients and 15 healthy controls, we identified 15 metabolites significantly associated with PD, with 11 reported as potential biomarkers for the first time. Key disrupted metabolic pathways include caffeine metabolism, arachidonic acid metabolism, and primary bile acid biosynthesis. Our machine learning model demonstrated high accuracy, with the Rotation Forest boosting model achieving 94.1% accuracy in distinguishing PD patients from controls. It is based on three new PD biomarkers (downregulated: 1-lyso-2-arachidonoyl-phosphatidate and hypoxanthine and upregulated: ferulic acid) and surpasses the general 80% diagnostic accuracy obtained from initial clinical evaluations conducted by specialists. Besides, this machine learning model based on a decision tree allowed for visual and easy interpretability of affected metabolites in PD patients. These findings could improve the detection and monitoring of PD, paving the way for more precise diagnostics and therapeutic interventions. Our research emphasizes the critical role of metabolomics and machine learning in advancing our understanding of the chemical profile of neurodegenerative diseases.

## Introduction

Parkinson’s disease (PD) is an age-related neurodegenerative disorder, currently affecting over 8 million individuals worldwide.^1^ It is primarily characterized by the loss of dopaminergic neurons associated with α-synuclein deposition, a protein implicated in various neurodegenerative processes.^2,3^ PD manifests through distinctive motor symptoms such as rest tremors, rigidity, slow movement, postural instability, and gait disturbances.^4^ The nonmotor symptoms include depression, gastrointestinal dysfunction, fatigue, and sleep disturbances.^5^ Consequently, PD is a chronic, progressive disorder affecting motor function, cognition, and the psychiatric state.

The diagnosis of PD predominantly depends on clinical assessment supplemented by neuroimaging. However, imaging modalities are expensive, sometimes inaccessible for some patients, and insufficient on their own to confirm a diagnosis, as findings can overlap with other neurodegenerative conditions. Moreover, patient misdiagnosis still occurs at least up to one-quarter of the time, especially when only clinical assessment is available.^6−9^ Thus, there is intense interest in blood-based biomarkers for PD diagnosis. While neuroimaging provides structural insights, fluid biomarkers could enable earlier diagnosis, dynamic monitoring of disease progression, and objective measurable end points for therapeutic trials.^4,6,9−11^

Metabolomics is an emerging field of scientific research that aims to study the metabolome of a given sample, such as cells, fluids, tissue, plants, and microorganisms.^7,12^ The nontargeted analysis, also known as untargeted metabolomics, allows for the evaluation of the majority of the metabolites present in a sample, facilitating the potential identification of diagnostic or prognostic biomarkers as well as potential drug targets. Moreover, untargeted metabolomics can be used to seek a better understanding of the pathophysiology of different diseases.^13^ This approach of the metabolome relies on highly sensitive analytical techniques, such as ultraperformance liquid chromatography coupled to an electrospray ionization source and quadrupole time-of-flight (UPLC-ESI-QTOF).^12,14^

Mass spectrometry (MS) is one of the main analytical techniques used in clinical metabolomics analysis, and when coupled with chromatographic separation techniques, as in the case of UPLC-ESI-QTOF, it offers higher sensitivity and greater resolution for the analysis of complex mixtures of metabolites.^3,15^ Recently, MS-based metabolomics strategies have been used to discover biomarkers for PD.^4,6,11,16^ However, despite promising results, no biomarker or set of biomarkers has been approved for clinical use yet. While there is still a lack of consensus on the most reliable and specific biomarkers for PD, blood is readily available, and it has a minimally invasive nature when compared to other relevant samples, such as the cerebrospinal fluid. Therefore, serum biomarkers provide reliable metabolic phenotypes of several organs, including the central nervous system, which may help to understand the pathogenesis and pathophysiology of PD.^6,16^

This study utilizes UPLC-ESI-QTOF in untargeted metabolomics to detect affected metabolic pathways in PD patients compared to those in healthy controls, aiming to identify potential PD biomarkers. Such insights are anticipated to deepen our understanding of PD’s metabolic dysfunctions and pave the way for effective blood diagnostic markers and therapeutic strategies. Notably, this is the first serum metabolomics investigation using a Brazilian PD human cohort. By focusing on serum biomarkers and altered metabolic pathways, this research seeks to contribute to future studies regarding earlier blood PD diagnosis and monitoring as well as to come across the potential for further development of targeted pathway treatments. Overall, this study lays the groundwork for expanded metabolomics research to elucidate PD metabolic dysfunction and translate these findings into clinically valid PD biomarkers.

## Results and Discussion

### Data Processing

**Figure 1:**
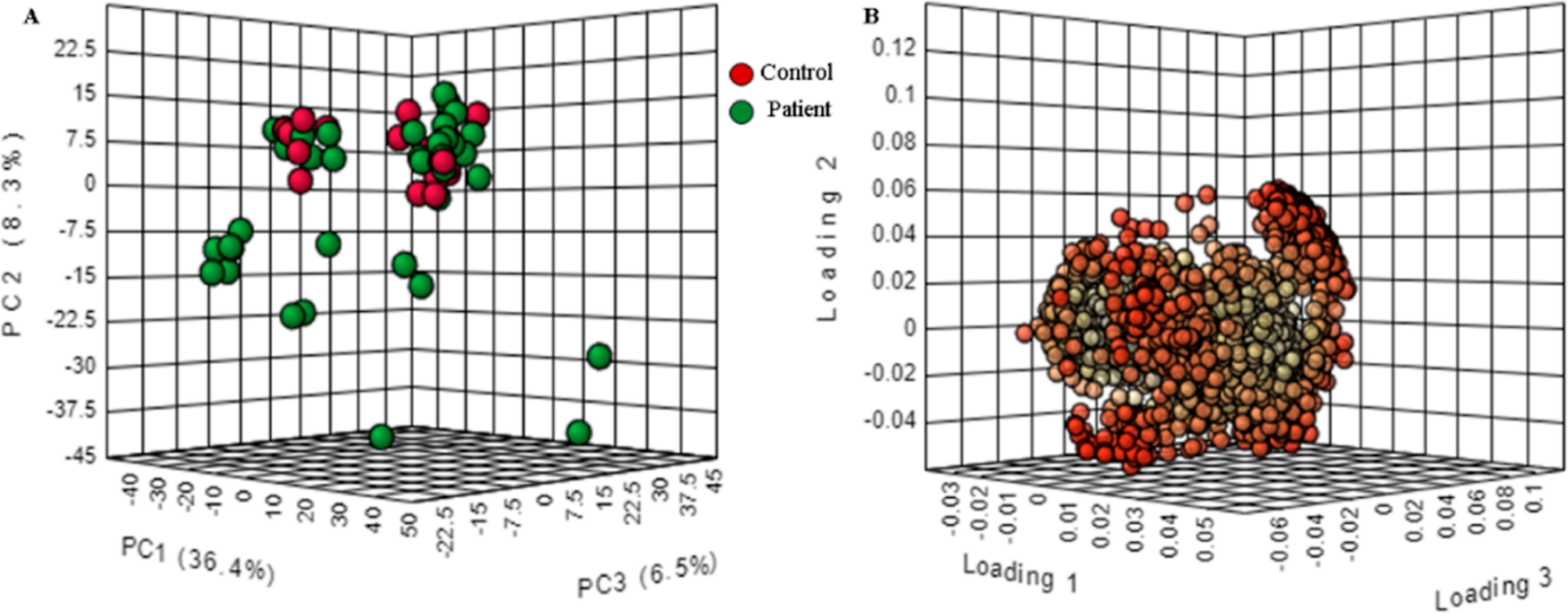
Unsupervised multivariate statistical analysis of PCA. (A) 3D score plot of PCA of samples, Hotelling’s ellipse = 95%, *R*^2^ = 0.57, and four components. The colors indicate the groups’ control (red) and patient (green). (B) Loading plot comprising all the variables of serum samples from patient and control groups.

**Figure 2:**
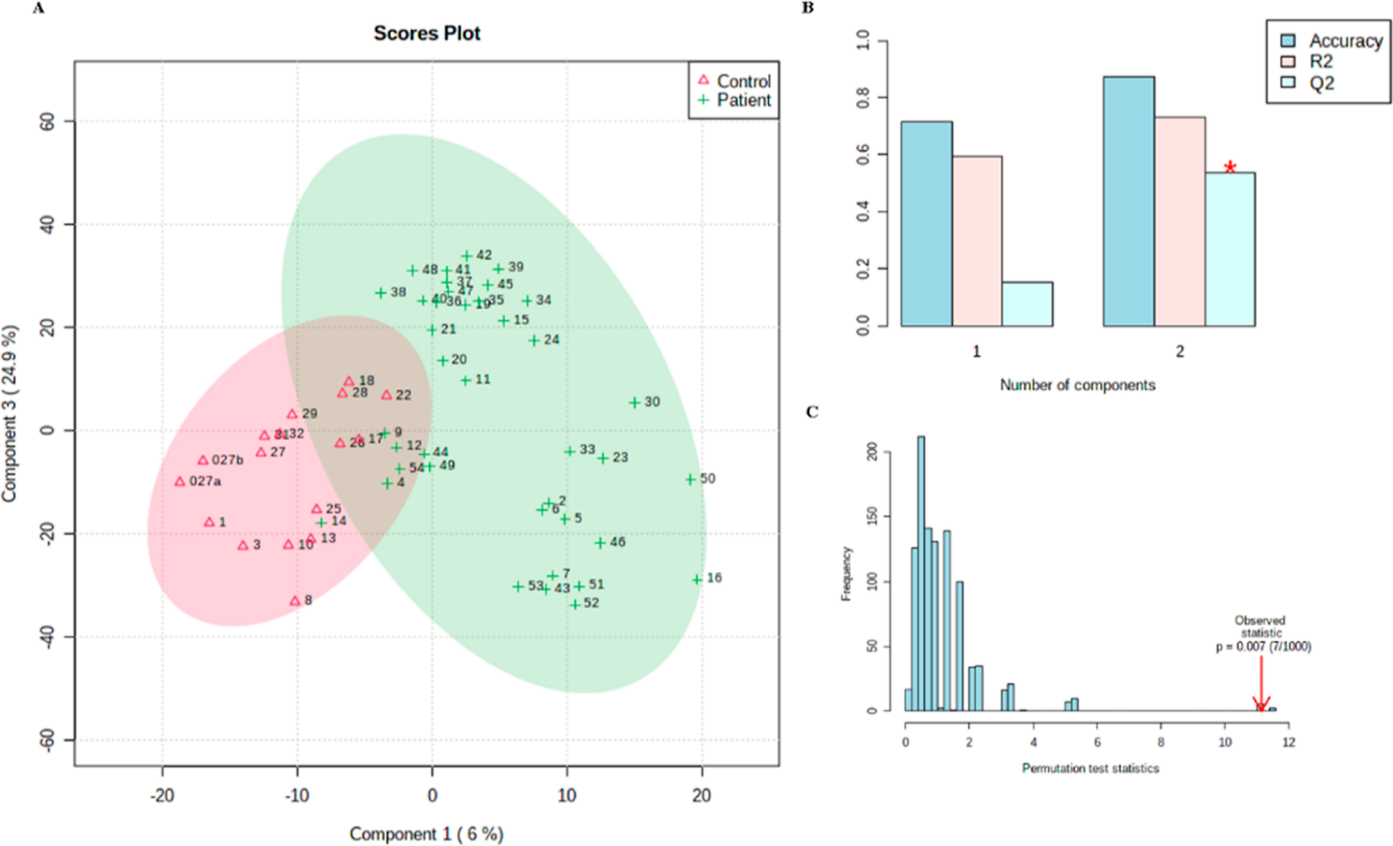
Multivariate statistical analyses of the UPLC-ESI-QTOF metabolomics data between PD patients and control groups. (A) 2D score plot of PLS-DA. (B) Cross-validation chart: performance of accuracy, *R*^2^, and *Q*^2^, with two components, *R*^2^ of 0.73, *Q*^2^ of 0.54, and accuracy of 0.875. (C) 1000× permutation test with *p* value < 0.05.

**Figure 3:**
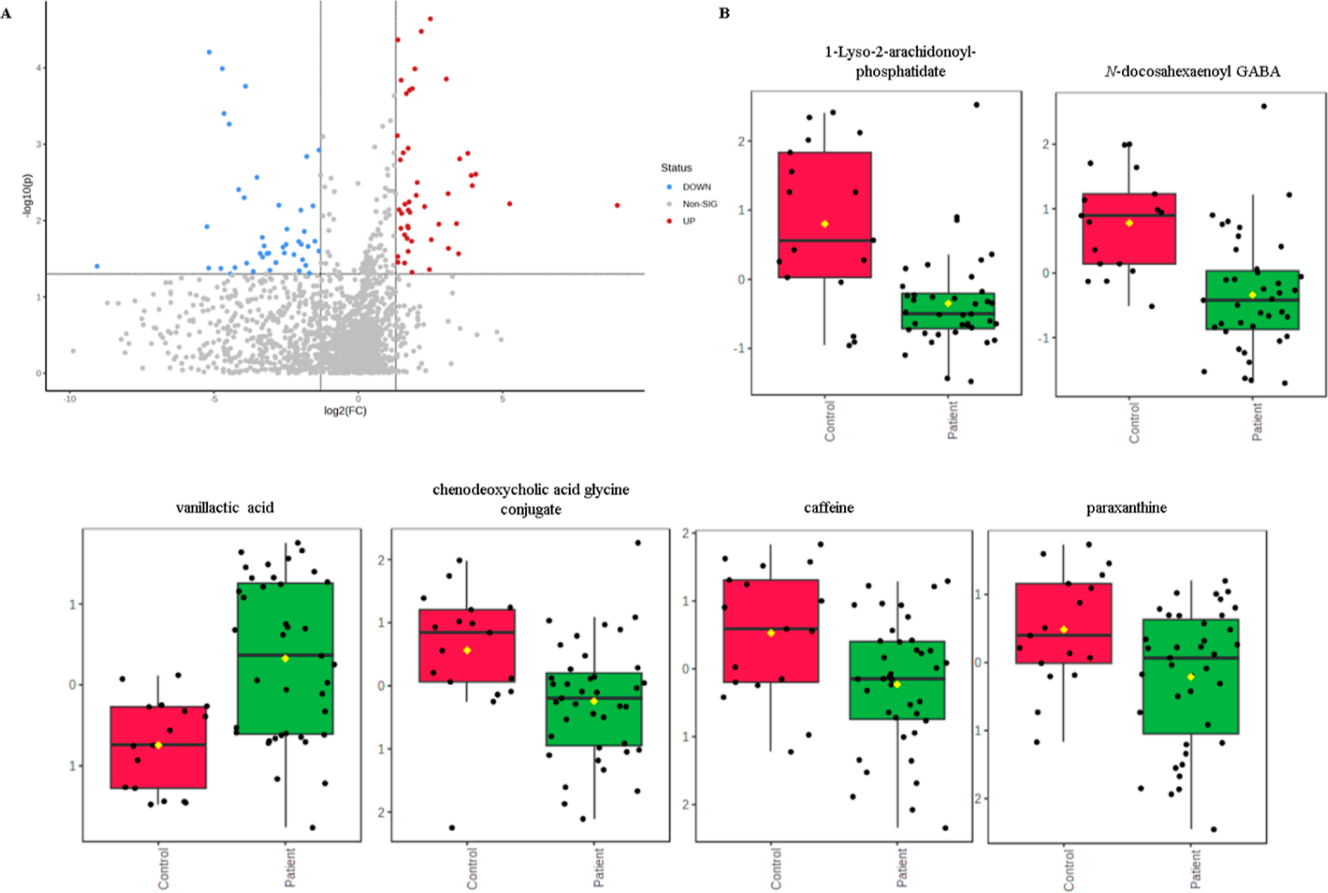
Univariate statistical analyses of the UPLC-ESI-QTOF metabolomics data from PD patients and control groups. (A) CVP. (B) Boxplots of the six most significant metabolites (*p* < 0.05) in the analysis of *t*-test results comparing the two groups (control: red boxes and patients: green boxes). The *x*-axis shows the specific metabolite and the *y*-axis is the normalized peak intensity.

The data set obtained through the UPLC-ESI-QTOF was processed using the MS DIAL software, resulting in the detection of 1923 features [retention time (RT) and *m*/*z* pair] in the positive mode and 1211 features in the negative mode, as a total of 3134 chemical features. The resultant data matrix underwent multiple normalization and statistical techniques, including *t*-tests, principal component analysis (PCA), volcano plots, frequency distribution, and bar plots, to support multivariate partial least-squares regression-discriminant analysis (PLS-DA) modeling and decision trees (Figures 1–3, Supporting Information, and attached data in 10.5281/zenodo.10960177). The analysis of results of the analytical replicates (blank and sample 27) showed reproducibility of their features obtained on the injections at the beginning, middle, and end of the UPLC-ESI-QTOF batch. After the data treatment, the peaks of replicates injected at the beginning, middle, and end of the UPLC-ESI-QTOF batch still superposed, and the features were as original. Thus, this analysis showed that the data processing is adequate.^12,14,17^

### Untargeted Metabolomics and PD’s Biomarkers

PCA is an unsupervised multivariate statistical analysis used to obtain an overview of sample distribution and a primary evaluation of the presence of potential outlier samples for a range of different types of metabolomics studies. In general, it is the first choice for analyzing the overall composition of any data set in a metabolomics study.^12,17,18^ The multivariate statistical analysis of the PCA, obtained with four components and an *R*^2^ value of 0.57, provided this initial overview of our metabolomics data set. This *R*^2^ value indicates that this unsupervised multivariate statistical analysis was well adjusted since values of *R*^2^ > 0.5 are considered significant in complex metabolomics data analysis.^12,14,19^ The PCA scatter plot (Figure 1) demonstrated significant clustering of control samples, although there was some overlap with part of the PD samples. Additionally, a clear dislocation of several PD samples in a more widespread clustering suggested potentially distinct metabolic profiles. After analysis of the loading plot, it confirmed and suggested discriminant features among the groups evidenced at the bottom part of the plot, more intensely red colored.

Thus, while the PCA revealed a partial separation between PD and healthy groups, some samples overlapped. This likely reflects the intricate metabolic variations between the groups and the complexity of the metabolomics data. Despite not achieving perfect discrimination, the analysis provided an invaluable first glance at the data set. It has also indicated that there were no outlier samples outside the 95% Hotelling’s ellipse, which relates to the general pattern of the metabolites and the chemical space in the metabolic profile of PD patients and healthy controls.

Furthermore, the supervised multivariate statistical analysis PLS-DA distinguished PD patients from healthy controls based on their serum metabolic profiles. PLS-DA has effectively separated the groups using a model of only two components and by exhibiting a *R*^2^ of 0.73, a *Q*^2^ of 0.54, and an accuracy of 0.875. Model validity was confirmed via 1000 permutation tests (*p* < 0.007; Figure 2). Further, variable important in projection (VIP) scores pinpointed 603 out of 2985 features significantly different (VIP > 1) between the groups marking the metabolic signature of PD patients. From those features, 15 statistically significant metabolites with high VIP values could be annotated (Table 1). For the univariate statistical analysis, the classical volcano plot (CVP) displayed the statistical significance from *t*-tests versus the fold change (FC) magnitude between the two evaluated groups. This approach helps to visualize multidimensional data effectively. The CVP analysis found 52 features exhibiting *p* < 0.05 and FC > 1.5 (Figure 3).

**Table 1:** List of 15 Metabolites Annotated That Differentiate Healthy Subjects (Control Group) from PD Patient Group^a^

biomarkers	pathway	molecular formula	observed *m*/*z*	MS^2^	adducts	mass error (mDa)	RT (min)	VIP	coef.	AUC	log 2 (FC)	*p* value	group	regulation in the PD group
1-lyso-2-arachidonoyl-phosphatidate (LPA)	arachidonic acid metabolism	C_23_H_37_O_7_P	457.235	309.27	[M + H]^+^	–0.830	6.475	3.288	100	0.757	2.496	<0.0001^c^	control	downregulated
*N*-docosahexaenoyl GABA	GABA metabolism	C_26_H_39_NO_3_	414.302	173.13	[M + H]^+^	–0.630	5.637	3.193	90.107	0.771	1.372	<0.0001^c^	control	downregulated
vanillactic acid	dopamine metabolism	C_10_H_12_O_5_	211.06	151.04	[M – H]^−^	0.397	1.841	3.054	89.776	0.947	–4.709	0.0049^c^	patient	upregulated
ferulic acid	phenolic compound metabolism	C_10_H_10_O_4_	195.066	177.05	[M + H]^+^	–0.126	1.381	2.964	89.59	0.644	–3.908	<0.0097^b^	patient	upregulated
3-*O*-methyl-a-methyldopa	dopamine metabolism	C_10_H_13_NO_4_	210.077	193.05	[M – H]^−^	0.260	1.378	2.817	83.898	0.923	–4.652	0.0164^c^	patient	upregulated
hypoxanthine	caffeine metabolism	C_5_H_4_N_4_O	137.046	69.1	[M + H]^+^	–0.213	0.663	2.380	69.043	0.623	2.043	0.0001^c^	control	downregulated
chenodeoxycholic acid glycine conjugate	primary bile acid biosynthesis	C_26_H_43_NO_5_	450.321	430.3	[M + H]^+^	–0.0009	3.895	2.307	69.72	0.709	1.476	0.0004^c^	control	downregulated
caffeine	caffeine metabolism	C_8_H_10_N_4_O_2_	195.089	138.06	[M + H]^+^	–0.350	2.024	2.167	64.615	0.594	1.788	0.0081^c^	control	downregulated
2-trans,4-cis-decadienoylcarnitine	carnitine metabolism	C_17_H_29_NO_4_	312.218	85.028	[M + H]^+^	–0.570	3.356	1.987	59.403	0.567		0.0146^c^	control	downregulated
pipecolic acid	lysine degradation metabolism	C_6_H_11_NO_2_	130.086	84.08	[M + H]^+^	–0.045	0.655	1.987	35.951	0.519		0.0072^c^	control	downregulated
paraxanthine	caffeine metabolism	C_7_H_8_N_4_O_2_	181.072	124.05	[M + H]^+^	–0.200	1.684	1.985	57.87	0.561	1.603	0.0343^c^	control	downregulated
15-HETE	arachidonic acid metabolism	C_20_H_32_O_3_	319.228	301.22	[M – H]^−^	0.368	7.669	1.590	49.749	0.543		0.0204^c^	control	downregulated
taurodeoxycholic acid	primary bile acid biosynthesis	C_26_H_45_NO_6_S	498.289	496.27	[M – H]^−^	0.270	5.108	1.263	36.378	0.605	1.227	0.0017^c^	control	downregulated
12-KETE	arachidonic acid metabolism	C_20_H_30_O_3_	319.227	91.054	[M + H]^+^	0.430	5.249	1.071	35.715	0.582		0.0277^c^	control	downregulated
15-HPETE	arachidonic acid metabolism	C_20_H_32_O_4_	335.222	317.21	[M – H]^−^	0.683	5.341	1.052	33.119	0.572		0.0459^c^	control	downregulated

a For each metabolite was given the biomarker annotation, the involved metabolic pathway, molecular formula, *m*/*z* value, MS^2^ main fragment, adducts, mass error, RT, VIP value, coefficients, areas under the curves (AUC) value, *p*-value, correlated group, and regulation in the PD.

b *p*-value obtained by the *t*-test.

c *p*-value obtained by the Mann–Whitney test. *p* < 0.05 indicates significant differences between the PD patients and control groups. log 2 (FC) values > 1 indicate a significantly higher magnitude. VIP—variable important in projection. FC—fold change. AUC—area under the curve. Coef—correlation coefficients. RT—retention time. MS^2^—high energy MS^E^ spectra.

The combined results from multivariate and univariate studies have corroborated the presence of metabolites responsible for differentiating PD patients from healthy controls (Table 1). These findings suggest that there are key metabolites that are contributors to the metabolic distinction between PD patients and healthy controls. While multivariate approaches consider multiple variables simultaneously, univariate methods assess one variable at a time. Each offers unique advantages and disadvantages, and thus combining multivariate and univariate statistical analyses can provide complementary insights. In this context, the consistent univariate data analyses results have further corroborated the previous multivariate analyses results and strengthened the confidence and robustness of the annotated metabolites that were statistically indicated as the most important for differentiating PD patients from the healthy control group.^20,21^

When compared to the literature, for the 15 annotated metabolites correlated with the PD group, caffeine (VIP = 2.17) and paraxanthine (VIP = 1.99) were consistently reported as downregulated in PD studies across diverse populations, including Japan,^22^ North America, Europe, Asia, and North Africa^9,23^, similar to what was observed in our research (Table 1). In addition, vanillactic acid (VIP = 3.05), identified in our study as upregulated, aligns with the findings from the research conducted within the Chinese population.^16^ Interestingly, increased serum vanillactic acid might hint at a potential impact of levodopa on dopamine (DA) metabolism and turnover, as the levels of vanillactic acid could also be altered by the levodopa degradation metabolism.^24^ However, further research is needed to elucidate the potential role of vanillactic acid in PD progression under and without the use of levodopa treatment. Thus, our findings corroborate previous literature and support evidence for these metabolites across different worldwide populations. Contrastingly, our data on hypoxanthine was also downregulated, which deviates from the existing literature,^25^ signaling a nuanced understanding of its regulation in PD and the need for further investigation.

Besides these three known metabolites affected in PD patients, 12 other metabolites were detected in different levels between normal and PD groups: 1-lyso-2-arachidonoyl-phosphatidate (LPA), *N*-docosahexaenoyl GABA, ferulic acid, 3-*O*-methyl-a-methyldopa (3-OMD) (not a biomarker), chenodeoxycholic acid glycine conjugate, 2-*trans*,4-*cis*-decadienoylcarnitine, pipecolic acid, paraxanthine, 15-hydroxyeicosatetraenoic acid (15-HETE), taurodeoxycholic acid, 12-keto-eicosatetraenoic acid (12-KETE), and 15-hydroxyeicosatetraenoic acid (15-HPETE).

From these compounds, 3-OMD (VIP = 2.82) was found to be upregulated. Considering that our PD cohort was undergoing levodopa therapy, the elevation of 3-OMD aligns with expectations, as it is a primary metabolite in the pharmacokinetics of levodopa.^26^ The PLS-DA association of 3-OMD with PD patients is, therefore, reliable and expected. In addition, the detection of elevated 3-OMD indeed underscores the power of metabolomics in detecting significant shifts in metabolic pathways, influenced by both disease pathology and pharmacological interventions. Thus, 11 metabolites were potentially novel detections in blood/serum as potential PD biomarkers, exhibiting either upregulation or downregulation (Table 1). Our findings not only align with but also expand upon the existing knowledge on PD biomarkers, proposing new hypotheses for metabolic changes and directions for future research related to biomarker-based diagnosis.

Besides, we rigorously annotated biomarkers following the Metabolomics Standards Initiative (MSI) recommendation, setting our research apart from some studies in the literature^22,27^ that omit important detailed analytical descriptions, such as details of MS acquisition and annotation procedure. Our approach goes beyond solely monoisotopic mass comparison, incorporating extensive mass spectra interpretation for more robust metabolite annotation and also more reliable findings. Importantly, our results, besides annotating some biomarkers for the first time, also align with some metabolites pointed out in relevant previous research, offering vital consistency for continuous scientific improvements in the field. This alignment provides a significant scientific contribution toward refining PD diagnosis, although the methodical approach is essential for advancing our understanding and diagnostic accuracy of this complex neurological condition, such as PD.

The high accuracy of the PLS-DA model and the novel findings from this study contribute to the growing body of evidence supporting the use of serum biomarkers in PD diagnosis and monitoring. Future research should focus on validating these biomarkers in larger cohorts and exploring their potential in clinical applications. Thus, to solidify the reliability of our findings, it is imperative to conduct further validation studies with more genetically heterogeneous human cohorts and include diet investigation. The existing literature highlights the significant impact of various factors, including population demographics, dietary patterns, genetic variability, and the stages of disease progression on the profile of PD.^28,29^ Thus, further research is essential to ensuring the robustness and applicability of the annotated biomarkers across diverse PD populations.

### Metabolic Pathways Enrichment Analysis

**Figure 4:**
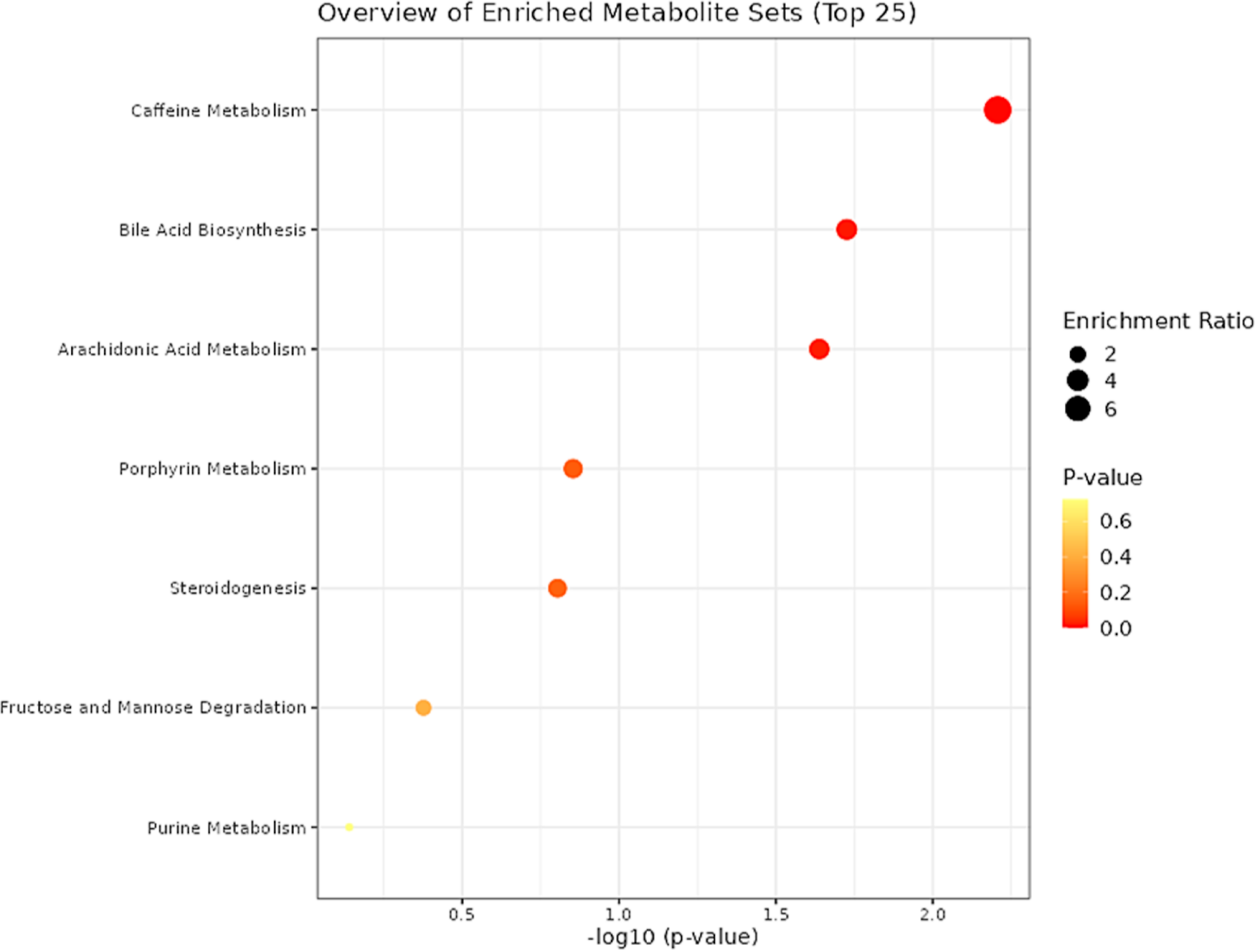
Mummichog analysis pointed out metabolic pathways affected in PD. The *y*-axis represents the main statistically significant metabolic pathways, and the *x*-axis represents the relative distribution of enriched pathways according to their raw logarithm *p*-values. The larger and reddish circles represent the most reliable affected pathways.

Employing Mummichog enrichment analysis to examine the significantly altered metabolites identified within the PD cohort compared to healthy controls, our study has elucidated the perturbation of several key metabolic pathways. Specifically, we observed disruptions in the caffeine metabolism, the biosynthesis of primary bile acids (BA), and the arachidonic acid (AA) metabolism (Figure 4).

Most pathways affected by the PD showed marked downregulation compared with the controls (Figure 4). Thus, targeted investigation of these pathways may uncover novel diagnostic and prognostic biomarkers, as well as opportunities for therapeutic intervention. The caffeine [*p*-value = 0.0081, Cohen’s *D* = 0.79, and false positive rate (FPR) = 0.054] and some of its main metabolites, such as paraxanthine (*p*-value = 0.0343, Cohen’s *D* = 0.60, and FPR = 0.173), were significantly lower in PD patients compared to the controls. It is well known that caffeine is absorbed from the small intestine and primarily metabolized by CYP1A2 and CYP2E1 in paraxanthine, theobromine, and theophylline, which are three dimethylxanthines.^30^

Despite the lack of dietary data for the individuals in this study, existing literature indicates a reduction in caffeine and its metabolites in PD patients compared to healthy controls, independent of daily caffeine intake.^22,30,31^ Recent studies found that moderate to advanced PD patients had lower blood caffeine levels compared with controls, while early stage PD patients showed no difference. The cause of this caffeine decrease is debated. One hypothesis states that the level of binding between serum albumin and caffeine decreases with disease progression. The increased free caffeine favors its metabolization, resulting in lower blood concentrations.^31,32^ Furthermore, there are shreds of evidence from animal model studies in the literature, showing that caffeine may reduce the risk of developing PD,^33,34^ and in humans, salivary caffeine decrease was correlated with PD progression.^31^ PD patients with lower caffeine levels were more associated with increased disease severity and the presence of motor impairments. Saliva testing provides an easy and noninvasive sample for PD patients’ clinical feature correlation and potential diagnosis,^31^ which additionally corroborated our serum analysis results.

Different research on the topic suggested the presence of a neuroprotective effect of caffeine, by acting as an inhibitor of the A2A adenosine receptor.^22^ This receptor is colocated in the striatum and associates with DA D2 receptors. It has also been shown that in animal models of DA dysregulation, A2A antagonists facilitate DA receptor signaling, normalizing motor function.^35^ Caffeine also has antioxidant and anti-inflammatory properties that may protect against oxidative damage and inflammation in the brain—two processes implicated in neurodegeneration.^34^ Overall, current evidence together indicates that caffeine’s metabolism in PD patients is dysregulated, while caffeine as a metabolite seems to have important neuroprotective effects.

Our findings indicate that the downregulation of caffeine and its metabolites has potential as a diagnostic biomarker for PD, regardless of caffeine being an exogenous metabolite. This possibility was not initially obvious. However, it appears highly promising due to the low costs for implementation and scaling. Further research is needed to fully understand the implications and determine whether this will be a viable and reproducible complementary diagnostic approach. Besides, further studies, including detailed dietary analysis, are crucial since biomarkers for PD, such as ferulic acid indicated by our metabolomics analysis, may originate exogenously from the human diet, similarly to caffeine.^36^ Thus, ferulic acid, as caffeine, is commonly found in dietary sources and is also being studied for its pharmacological potential in neurodegenerative diseases due to its strong antioxidant and neuroprotective properties.^36^ In this study, increased levels of ferulic acid were observed in the serum of PD patients. These findings require further investigation, and the role of ferulic acid in PD remains promising, underscoring the need for further research to fully elucidate its potential as a biomarker or a therapeutic agent in the management of PD.^36^

The synthesis of BA was the second most affected pathway with statistically significant changes observed. BA is synthesized from cholesterol through cytochrome P450 enzymes located in hepatocytes. They act primarily to solubilize dietary lipids and fat-soluble vitamins and influence metabolic processes by acting as signaling molecules that bind membrane receptors. BA also has secondary functions such as steroid hormone regulation.^37^ BA has been reported as altered in PD. Some studies in animal models and human tissue samples showed increased secondary BA levels, while others evidenced reduced levels of primary BA.^16,38−40^

In our study, chenodeoxycholic acid glycine conjugate (*p* = 0.0004, Cohen’s *D* = 0.88, and FPR = 0.004), a specific primary BA, was downregulated. Cholic acids are the only BA synthesized in the human organism, which impacts fat digestion and energy metabolism signaling.^41^ When BA is released from the gallbladder, most are reabsorbed in the ileum and transported back to the liver, and the remaining BA is metabolized by the intestinal microbiota into secondary BA.^38^ The brain and gut have bidirectional communication that has been associated with neurodegenerative diseases, including PD.^38,41^ The conversion of primary BA to secondary BA by the gut microbiota is an important process in regulating the balance in the organism. According to our results, the decrease in the primary BA might implicate the upregulated secondary BA reported in the literature due to gut microbiota imbalance. Thus, alterations in the gut microbiome can lead to a discrepancy in the BA profile, which has been associated with inflammatory diseases like PD. BA may also relate to PD through neuroinflammation and apoptosis theories since chenodeoxycholic acid (CDCA) can bind the protein-coupled TGR5 receptor to reduce the apoptosis process.^42,43^

A recent study using an MPTP mouse model of PD observed that mice treated with BA after MPTP injury showed improved motor function, movement initiation, and tremor correction differently from those of mice treated with MPTP alone. Levels of parkin, a ubiquitin ligase involved in mitochondrial biogenesis, were also maintained in BA-treated mice differently from those of the MPTP alone group. These results demonstrate the neuroprotective activity of the BA in this PD model.^40^ Another study showed BA antioxidant activity in this model by preventing reactive oxygen species and upregulating glutathione peroxidase and heme oxygenase-1.^39^ While the BA-PD relationship remains unclear, these findings suggest a possible link between gut microbiome alterations, BA metabolism changes, and PD. Further research is needed to better elucidate the gut microbiome and BA metabolism as an altered metabolite pathway in PD.

The AA metabolism pathway was also significantly altered in PD patients. The AA is metabolized into downstream products, including HETEs, prostaglandins, and leukotrienes. These mediators modulate inflammation, oxidative stress, and other processes implicated in PD pathogenesis.^44−47^ While higher HETE levels might be expected in PD due to inflammation, our and other recent metabolomics studies found downregulated HETEs and other AA-derived metabolites.^3,48^ We observed reduced 12-KETE (*p* = 0.0277, Cohen’s *D* = 0.49, and FPR = 0.17), 15-HETE (*p* = 0.0204, Cohen’s *D* = 0.49, and FPR = 0.15), and LPA (*p* < 0.0001, Cohen’s *D* = 1.3519, and FPR = 9 × 10^–4^) in PD patients. This suggests the dysregulation of lipid mediators and the AA cascade in PD. The metabolomics results suggest that AA metabolism via the lipoxygenase enzyme (LOX) pathway is altered in PD via downregulated metabolites.

Notably, 15-HETE and 12-KETE have exhibited neuroprotective and anti-inflammatory effects in models of brain ischemia and neurodegeneration.^44,45^ However, few human studies have consistently reported AA pathway downregulation in PD.^3,48^ While the mechanisms underlying AA alterations in PD remain unclear, our results and other emerging evidence implicate dysfunction of this pathway in PD pathogenesis. Further elucidation of AA metabolism changes in PD is warranted to determine their significance and potential as therapeutic targets.

### Prediction Models

Investigating PD through the lens of computational chemistry and machine learning algorithms, our study employed the J48 decision tree boosted by the Rotation Forest (RF) ensemble method to analyze and predict the metabolomic profiles of PD patients. The J48 model demonstrated 88.2% accuracy on the external test set and 86.5% accuracy on the internal training set. This indicates good model performance with a reasonable balance to avoid overfitting. Additionally, metrics like sensitivity and specificity highlight the model’s ability to accurately detect true positives (patients) and true negatives (controls).

Notably, the RF ensemble method significantly enhanced the performance of the J48 model, achieving a superior accuracy of 94.1% for the J48 external test set. This enhancement in performance illustrates the model’s advanced capability in differentiating patients from controls, offering promising directions for diagnosis and personalized treatment strategies in PD (Table 2). The integration of multiple decision trees in the RF method contributed to this enhanced performance.

**Table 2:** J48 Decision Tree and Ensemble Method RF J48 Performance Metrics^a^

ensemble methods	metrics	accuracy (%)	Cohen’s κ	sensitivity	specificity	precision	recall	*F*-measure
J48	accuracy	94.6	0.871	1	0.926	0.833	1	0.909
	external validation	88.2	0.679	0.600	1	1	0.600	0.750
	internal validation	86.5	0.668	0.800	0.889	0.727	0.800	0.762
J48-Rotation Forest	accuracy	100	1	1	1	1	1	1
	external validation	94.1	0.850	0.800	1	1	0.800	0.889
	internal validation	91.9	0.788	0.800	0.963	0.889	0.800	0.842

a Compares accuracy, Cohen’s κ, sensitivity, specificity, precision, recall, and *F*-measure.

**Figure 5:**
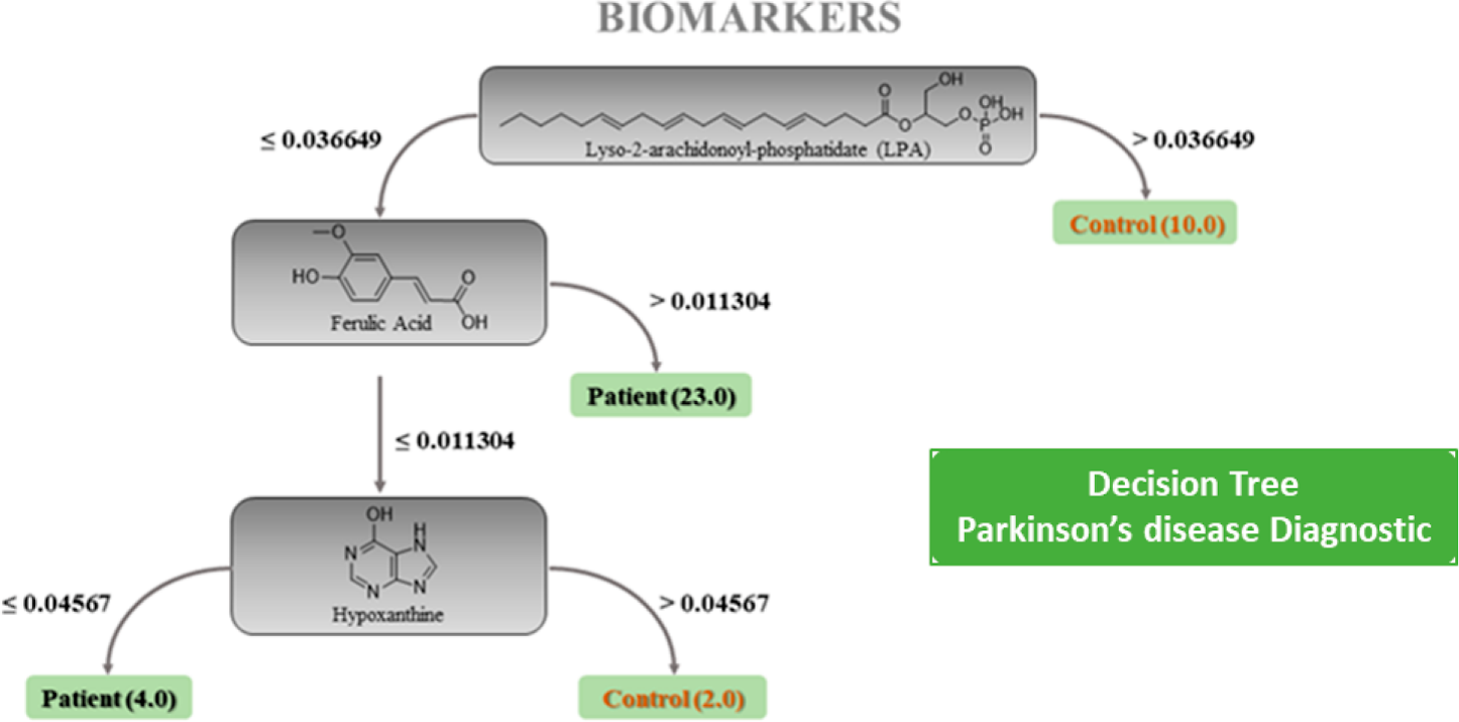
Decision tree model (J48) illustrating serum biomarkers PD diagnosis. The decision nodes are based on the intensity levels of LPA, ferulic acid, and hypoxanthine, leading to the classification of subjects as patients or controls. Values at the arrows from each node represent intensity level thresholds that separate the patient group from the control group. Leaf nodes (in green) display the number of subjects classified as patients or controls based.

The exploration of the key metabolites and advanced predictive modeling can offer a fascinating glimpse into the future of PD diagnostics. The key metabolites pinpointed by the developed decision tree (J48) prediction model included LPA, ferulic acid, and hypoxanthine, which together could play a crucial role in distinguishing between PD patients from healthy individuals (Figure 5). Remarkably, this is the first time in literature where the metabolites LPA (downregulated), ferulic acid (upregulated), and hypoxanthine (downregulated) have been associated with PD in serum metabolic profiles, potentially aiding in biomarker-based diagnosis.

This decision tree, structured like a simple flowchart, offers insights into the specific levels of metabolites, which most notably influence patient classification (Figure 5). Thus, satisfactorily, the RF-J48 model achieved a validated accuracy of 94.1% (external test), surpassing the traditional diagnostic accuracy (∼80%) derived from initial clinical evaluations by movement disorder specialists.^49^ Thus, the level of these metabolites could potentially contribute to future reliable biomarkers in PD diagnosis as it can support clinical diagnostics and contribute to minimizing potential misdiagnoses.

In summary, the decision tree proposed herein offers an approach to selecting and understanding the relationships between key metabolites in PD patients and healthy controls. The visual interpretability of the J48 decision tree enables easily understandable analysis, thereby guiding the overall decision-making logic based on metabolite profiling, leveraging untargeted metabolomics and machine learning tools to identify potential biomarkers for PD diagnosis.^50,51^ The RF ensemble technique significantly enhanced the J48 machine learning model accuracy by offering a reliable way to elevate predictive performance. These methods are known for their superior generalization abilities due to the integration of multiple trained models.^50,51^ This synergistic approach can not only enhance our diagnostic toolkit but also contribute to groundbreaking biomarker identification and prediction in modern PD metabolomics studies.

## Methods

### Patient Cohorts

Study participants included patients (*n* = 39, 19 men and 20 women, and mean age = 65.4 ± 12.6 years old) who were diagnosed with PD by neurologists from the University Hospital of Federal University of Juiz de Fora, Brazil, during regular outpatient visits between 2018 and 2019. Most of the patients were diagnosed using the Unified PD Rating Scale Part 3 (motor subscore), Schwab & England score, and Hoehn and Yahr scale to assess the severity of symptoms and disability associated with PD, including patients from stages 1 to 4. All PD patients were using levodopa as the main PD pharmacological approach. The healthy subjects (*n* = 15, 8 men and 7 women, and mean age = 55.6 ± 10.2 years old) to the control group were recruited based on the criteria of no diagnosis of neurological manifestations or gastrointestinal manifestations and no family history of neurological diseases. All participants in this study were volunteers, who provided written informed consent as approved by the research ethics committee of the University Hospital of the Federal University of Juiz de Fora advisory board of the National Health Council (CAAE no. 70592617.2.0000.5133). This study complies with the Declaration of Helsinki.

### Serum Sample Preparation

The materials used were 2 mL safe-lock microtubes (Eppendorf) and vacutainer tubes with clot activator 13 × 100 mm (BD Biosciences). The equipment used included a centrifuge 5403 (Eppendorf) and a lyophilizer (JJ Cientifica LJJ02). Nonfasting whole blood samples for analysis were collected from a peripheral vein of all participants in serum vacutainer tubes. The serum was separated by centrifugation at a rotation of 1000 rcf at room temperature for 5 min. Serum aliquots of 200 μL were taken into safe-lock microtubes, and 400 μL of acetonitrile (ACN) was added for protein precipitation. The mixture was vortexed for 10 s and centrifuged at 15,332 rcf for 10 min at 22 °C. The supernatant (400 μL) was transferred to a new microtube and placed in a SpeedVac instrument for 4 h to dry the organic solvent. The remaining aqueous phase was frozen and lyophilized (−48 °C). After complete drying, samples were resuspended in 100 μL of H_2_O/ACN (1:1, v/v) and centrifuged at the same conditions, and 80 μL of supernatant was transferred to an insert for further analysis in the UPLC-ESI-QTOF system.

### UPLC-ESI-QTOF Data Acquisition and Analysis for Untargeted Metabolomics

Solvents and reactants used were of high-performance liquid chromatography grade, including acetonitrile from Sigma-Aldrich (St Louis, MO, USA) and formic acid supplied by Sigma-Aldrich (St Louis, MO, USA). Ultrapure water was purified using a Millipore Milli-Q water purification system (Millipore, Bedford, MA, USA). The metabolomic analyses were realized by using UPLC-ESI-QTOF (Xevo-QTOF/MS, Waters). The column oven was kept at a temperature of 40 °C, and the mobile phases consisted of acidified Milli-Q water with 1% v/v formic acid (A) and ACN (B). The chromatographic runs were realized using a reversed-phase C18 ACQUITY UPLCHSS T3 ultra-analytical column (1.8 μm, 100 × 2.1 mm). The gradient elution method consisted of 10 min of total run with 0.5 mL/min of flow rate. The chromatography method includes an injection volume of 5 μL and was defined as 1% ACN and 99% H_2_O (0.1 min), 85% H_2_O and 15% ACN (7.5 min), 20% H_2_O and 80% ACN (8.5 min), 1% H_2_O and 99% ACN (8.6 min), and 99% H_2_O and 1% ACN (up to 10 min).

The ESI was operated in both negative and positive ionization modes. The MS^E^ technique, which is a data-independent acquisition technique, was used for the mass acquisition mode to allow for more complete coverage of MS fragments. The operating parameters of the equipment consisted of 40 V for cone voltage, 3.0 kV for capillary voltage, 30 L/h of cone gas flow, 300 °C desolvation temperature, 120 °C of source temperature, and 600 L/h for the desolvation gas flow. The mass scan range was set to 100–1000 *m*/*z*. Moreover, the MS data were collected in the centroid mode. The lock spray (200 pg/mL leucine enkephalin), identified by *m*/*z* 554.2622 (ESI^–^) and *m*/*z* 556.2768 (ESI^+^), was applied to calibrate the equipment.

The samples were analyzed in a randomized way, in which a replicate (sample 27) and a blank sample (pure ACN from sample preparation) were injected at the beginning, middle, and end of the chromatographic runs to ensure robustness for posterior data processing and analysis.^12,14,17^

### Data Processing

The UPLC-ESI-QTOF data was processed in MS DIAL software version 4.70.^52^ For data collection were used MS^1^ tolerance (0.05) and MS^2^ tolerance (0.1). For the peak detection, a minimum peak height (5000 amplitude) and mass slice width (0.1 Da) were set. The deconvolution parameters were defined as sigma window value (0.7) and MS/MS abundance cutoff (70 of amplitude). For the identification were used RT tolerance (10 min), accurate mass tolerance—MS^1^ (0.01), accurate mass tolerance—MS^2^ (0.05 Da), and identification score cutoff (80%). For alignment of data were used RT tolerance (0.3 min for the negative mode and 0.2 for the positive mode) and MS^1^ tolerance (0.015 Da for the negative mode and 0.025 for the positive mode). Adduct search for the data from mass detection in the positive mode included [M + H]^+^, [M + NH_4_]^+^ [M + Na]^+^, and [M + K]^+^. For the negative mode, it was [M – H]^−^, [M – H_2_O – H]^−^, [M + Cl]^−^, and [M + Br]^−^. The consistency of results from the blank and sample replicates was evaluated.^12,14,17^

### Multivariate and Univariate Statistical Analyses

The UPLC-ESI-QTOF data including the peak area and the features of each metabolite was processed using the MS DIAL software version 4.70. Analysis was performed for both positive and negative ionization modes and exported as a.csv file. The spreadsheet was imported through MetaboAnalyst 5.0 software (Montreal, QC, Canada) for statistical analysis. The data were normalized by the median, log transformation, and mean-centered scale before the analysis. Unsupervised statistical analysis using PCA was done to have an overview of the data set. The data was also analyzed by supervised statistical analysis, using the PLS-DA, to find potential biomarkers of PD.

The *R*^2^ and *Q*^2^ values were calculated to assess the model fitness and robustness of the PLS-DA model, respectively. Permutation tests (*n* = 100) were applied as an internal validation method to ensure the absence of overfitting.^53^ Furthermore, these two multivariate statistical analyses, PCA and PLS-DA, a univariate statistical method of CVP, were also performed. The CVP incorporates *p*-values from the *t* tests and FC magnitudes. Therefore, a metabolite was only considered statistically significant and a potential biomarker variable when having VIP value > 1 and *p*-value < 0.05, in the PLS-DA and CVP analyses, respectively.^17,19,54^

In addition, data normality was assessed using the Anderson–Darling, D’Agostino–Pearson, and Shapiro–Wilk tests. Numerical parameters, including mean, median, coefficient of variation, skewness, and kurtosis, were evaluated. Graphical analyses such as box plots and frequency distributions complemented numerical assessments. This comprehensive approach, combining statistical tests, numerical metrics, and graphical visualization, enabled rigorous evaluation of data normality assumptions required for subsequent analyses. Due to the observed non-normality of the data for the several metabolites in the raw data matrix, the Mann–Whitney test (nonparametric test) was used to assess the difference between the means of the case and control groups leading to a more confident *p*-value. To ensure the reliability of the observations, the statistical power of the test, size effect (Cohen’s *D*) (G*Power version 3.1.9.6., Heinrich Heine University Düsseldorf, Germany), and FPR (False Positive Rate Web Calculator, version 1.7, Longstaff, C. and Colquhoun D) parameters were considered and are available in the Supporting Information and the following link 10.5281/zenodo.10960177.

To assess the diagnostic potential of the identified biomarkers, receiver-operating characteristic (ROC) curves were generated, and the AUC were calculated based on the highest VIP and lowest *p*-values. The ROC and AUC analyses were used to evaluate the ability of individual metabolites to distinguish between PD patients and controls.^4^ The data spreadsheet of the metabolites selected on multivariate statistical analysis was imported into MetaboAnalyst 5.0 software (Montreal, QC, Canada) for analysis and was normalized by the median, transformed by log(base 10), and mean-centered scaled by Pareto before classical univariate ROC curve analyses were performed. This analysis provided valuable information about the accuracy of the identified biomarkers in discriminating between PD patients and controls.

### Annotation of the Compounds and Metabolic Pathway Analysis

Annotation was performed using the integrated library platforms and scientific chemical databases integrated into MS DIAL software, version 4.70. The processed data were directly exported to MS FINDER tool version 3.52 for the annotation of the compounds. To search for the potential presence of adducts, the cations Na^+^, K^+^, Mg^+^, and Ca^2+^ were included in the data analysis of positive mode, and the anions I^–^, Cl^–^, S^–^, and Br^–^ were included in the negative mode. The relative abundance of M + 2 isotopes was analyzed separately to confirm the possible presence or absence of adducts. The maximum allowable *m*/*z* error limit was set at 10 ppm. Metabolites were identified at level 2 confidence as established by the MSI, by analyzing the characteristic MS/MS fragments matched to reference spectra in the literature.^55−57^ Each generated molecular formula and MS^2^ spectra were manually verified against the HMDB database (The Human Metabolome Database, https://hmdb.ca/), ensuring that fragmentation patterns align with the suggested structure of the compound. Standardization of metabolite annotation enhances the reliability and depth of comparative chemical information related to the annotated metabolites.^58^

The metabolic pathway analysis was performed using Mummichog 3 and the enrichment analysis using MetaboAnalyst 5.0 software (Montreal, QC, Canada). For the analysis, default parameters were kept: mass accuracy of 10 ppm and pathway enrichment analysis with 1000 permutations. Metabolites indicated from the PLS model were used as input data to Mummichog 3 to evaluate the enrichment pathway compared to the *Homo sapiens* species database (PMDB and KEGG), yielding an empirical *p*-value for each pathway. Pathways with *p*-values < 0.05 were considered relevant.

### Prediction Models and Hyperparameter Tuning

The data set used to build prediction models included eight annotated metabolites with significant differences between the PD and healthy group in all statistical metrics (VIPs > 1 and *p* < 0.05): LPA, *N*-docosahexaenoyl GABA, ferulic acid, hypoxanthine, chenodeoxycholic acid glycine conjugate, caffeine, paraxanthine, and taurodeoxycholic acid. The data set was saved in.csv files and partitioned into training and test sets for further validation evaluations, using the KNIME 5.1 platform (https://www.knime.com).^49,59^ The node “X-Partitioner” was used to fragment the data into 70% for training (38 random patients and control samples) and 30% for testing (16 random patients and control samples). The internal validation was performed with the selection of 10 random groups (10 folds). For that, the nodes “X-Aggregator” and “Score” were used, respectively, to combine and save the outcomes of each validation step.^60^ The J48 decision tree algorithm (C4.5) from Weka software was applied to the training data to construct the machine learning decision trees and determine possible metabolites that could differentiate PD patients from healthy controls.^61,62^

In addition, hyperparameter tuning was performed to improve the performance of the J48 decision tree model. The parameter scanning in the tuning process was performed using hyperparameter optimization algorithms with cross-validation using “loops” nodes and using the search strategy brute force where all possible combinations of parameters are checked and the best one is returned.^63−65^ On top of that, the RF ensemble method was applied to the tuned J48 model due to its feature space rotation approach using PCA prior to classifier training. RF segments the decision space along both feature axes and varied orientations, offering advantages when all independent variables of the data set are continuous, such as our metabolomics data.^64,66,67^ Three key metrics were analyzed for validation models: accuracy, internal, and external validation.^65,68,69^ A range of parameters used are online available in Table S1 and Figure S1 at the Supporting Information and link 10.5281/zenodo.10960177.

## Conclusions

In conclusion, this study represents the first evaluation of a Brazilian PD cohort using untargeted metabolomics. The analysis revealed significant alterations in metabolic pathways related to caffeine metabolism, AA metabolism, and BA synthesis, alongside the annotation of 11 novel biomarkers previously unreported in the literature. Detailed data analysis and chemical confidence annotation levels were meticulously reported, laying the groundwork for developing novel optimized PD biomarker diagnostics. The literature accounts for factors such as population, genetics, disease progression stages, and diet, which can affect the PD metabolic profile. Thus, further validation of the pinpointed biomarkers using larger, diet-supervised, and more genetically diverse human cohorts is essential. Additionally, the use of machine learning based on decision trees provided a visual interpretable analysis of affected metabolites in patients with PD compared with healthy controls using a simple structured flowchart. The ensemble prediction models pointed out key new biomarkers, LPA and hypoxanthine (both downregulated), and ferulic acid (upregulated), which could discriminate PD and healthy human cohorts with an accuracy of 94.1% in the external set. This surpasses the ∼80% diagnostic accuracy achieved in initial clinical evaluations by specialists, highlighting the potential to reduce misdiagnoses of PD. Thus, our comprehensive investigation found statistically significant serum biomarkers and disrupted metabolic pathways that might aid in understanding PD pathogenesis. These findings not only shed light on the complex-associated metabolic dysregulations that can aid in diagnosis but also hint at novel therapeutic targets for further research. Moving forward, rigorous validation in clinical settings will be crucial to fully harnessing the potential of metabolomics analysis in refining PD diagnostic accuracy, prognosis, and management.

## Supporting Information Available

UPLC-ESI-QTOF data are deposited and available in the MS Interactive Virtual Environment (MassIVE) repository; data are under registry code MSV000095323 and can be accessed at the following link: ftp://massive.ucsd.edu/v08/MSV000095323/; another part of the Supporting Information can be accessed at the following link: 10.5281/zenodo.10960177; Mann–Whitney test results, data sets used for the J48 model development, split into complete, test, and training sets; outputs from metabolomics analysis performed using the Mummichog tool, presented in both negative and positive ionization modes; normality test results; volcano plot data; and each file is accompanied by its MD5 checksum to verify the integrity of the data (PDF)

## Abbreviations

3-OMD: 3-*O*-methyldopa
12-KETE: 12-keto-eicosatetraenoic acid
15-HETE: 15-dydroxyeicosatetraenoic acid
5-HPETE: 15-hydroxyeicosatetraenoic acid
AA: arachidonic acid
AUC: areas under the curves
BA: bile acids
CDCA: chenodeoxycholic acid
CVP: classical volcano plot
DA: dopamine
DIA: data-independent acquisition
FC: fold change
FPR: false positive rate
HPLC: high-performance liquid chromatography
LOX: lipoxygenase enzyme
LPA: 1-lyso-2-arachidonoyl-phosphatidate
MS: mass spectrometry
MSI: metabolomics standards initiative
PCA: principal component analysis
PD: Parkinson’s disease
PLS-DA: partial least-squares regression-discriminant analysis
RF: Rotation Forest
ROC: receiver-operating characteristic
RT: retention time
UPDRS: unified Parkinson’s Disease rating scale
UPLC-ESI-QTOF: ultraperformance liquid chromatography coupled to an electrospray ionization source and quadrupole time-of-flight
VIP: variable important in projection
