# Supplementary material for: Metabolomics Unveils Disrupted Pathways in Parkinson’s Disease: Toward Biomarker-Based Diagnosis
Source: ACS Chem Neurosci. 2024 Aug 23;15(17):3168–80. doi: 10.1021/acschemneuro.4c00355 (PMC11378289; doi:10.1021/acschemneuro.4c00355)
Supplement: Supplementary file 1 — cn4c00355_si_001.pdf [file cn4c00355_si_001.pdf]

Supporting information

Metabolomics unveils disrupted pathways in Parkinson's disease: towards biomarker-based diagnosis

Authors

Wanderleya Toledo dos Santos: Department of Pharmaceutical Sciences, Federal University of Juiz de Fora, Juiz de Fora 36036-900, Brazil. https://orcid.org/0000-0001-9693-8738, wanderleyatoledos@gmail.com

Albert Katchborian-Neto: Chemistry Institute, Federal University of Alfenas, Alfenas 37130-001, Brazil. https://orcid.org/0000-0003-2711-2881, albert_katchborian@hotmail.com

Gabriel Silva Viana: Chemistry Institute, Federal University of Alfenas, Alfenas 37130-001, Brazil. https://orcid.org/0000-0002-5450-9860, gabrielsilva.viana@sou.unifal-mg.edu.br

Miller Santos Ferreira: Chemistry Institute, Federal University of Alfenas, Alfenas 37130-001, Brazil. https://orcid.org/0000-0002-9723-0528, millersantosferreira@gmail.com

Luiza Martins: Faculty of Medicine, Federal University of Juiz de Fora, Juiz de Fora 36036-900, Brazil. https://orcid.org/0000-0002-5553-7628, martinslu091@gmail.com

Thiago Vale: Faculty of Medicine, Federal University of Juiz de Fora, Juiz de Fora 36036-900, Brazil. https://orcid.org/0000-0001-6145-9868, thiagocardosovale@hotmail.com

Michael Murgu: Waters Corporation, Barueri 06455-020, Brazil. https://orcid.org/0000-0002-7489-7637, michael_Murgu@waters.com

Danielle Ferreira Dias: Chemistry Institute, Federal University of Alfenas, Alfenas 37130-001, Brazil. https://orcid.org/0000-0001-9129-4734, danielle.dias@unifal-mg.edu.br

Marisi Gomes Soares: Chemistry Institute, Federal University of Alfenas, Alfenas 37130-001, Brazil. https://orcid.org/0000-0001-9221-9867, marisigs@gmail.com

Daniela Aparecida Chagas-Paula: Chemistry Institute, Federal University of Alfenas, Alfenas 37130-001, Brazil. https://orcid.org/0000-0003-2274-4919, daniela.chagas@unifal-mg.edu.br

Corresponding Author

Ana Claudia Chagas de Paula: Department of Pharmaceutical Sciences, Federal University of Juiz de Fora, Juiz de Fora 36036-900, Brazil. https://orcid.org/0000-0001-7998-0950, ana.chagas@farmacia.ufjf.br

Contents

1. Supplementary Data - Mann-Whitney Tests 3

2. Supplementary Figures and Tables 10

Table S1. Parameters and values evaluated for the J48 method using the loop optimization node. 10

Figure S1. "X-Aggregator" and "Score" nodes combined for the model validation step. 11

3. Dataset for J48 Model - Complete, Test, and Training Sets 12

Table S2. Complete J48 dataset model including selected VIPs 12

Table S3. J48 Test dataset model including selected VIPs. 14

Table S4. J48 Train dataset model including selected VIPs 15

4. Metabolomics Analysis with Mummichog. 16

Table S5. Data from negative mode used on Mummichog analysis. 16

Table S6. Data from positive mode used on Mummichog analysis. 18

5. Normality test results 21

Table S7. Control group normality tests results. 21

Table S8. Patient group normality tests results. 23

6. VIP (Variable Importance in Projection) and Mass-to-Charge Ratios (*m/z*). 25

7. Volcano Plot Data 29

Table S10. Volcano test 29

1. Supplementary Data - Mann-Whitney Tests

1-Lyso-2-arachidonoyl-phosphatidate (LPA)

Mann-Whitney test: *p*-value < 0,0001

Cohen's D: 1,3519

Power post-hoc (1 – β err prob): 0.9815

False positive risk: 9×10^{-04}

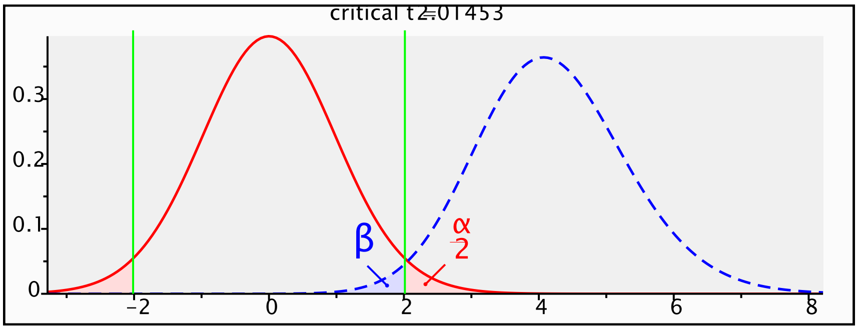

N-Docosahexaenoyl GABA

Mann-Whitney test: *p*-value < 0,0001

Cohen's D: 0.9452

Power post-hoc (1 – β err prob): 0.8075910

False positive risk: 0.0015

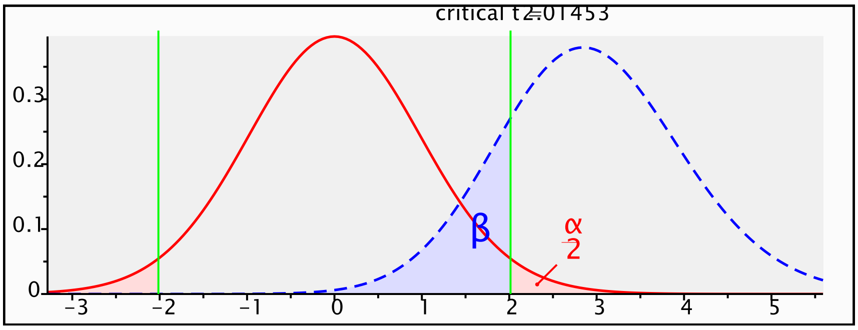

Vanillactic acid

Mann-Whitney test: *p*-value = 0,0049

Cohen's D: 1.1494240

Power post-hoc (1 – β err prob): 0.9305652

False positive risk: 0.0465

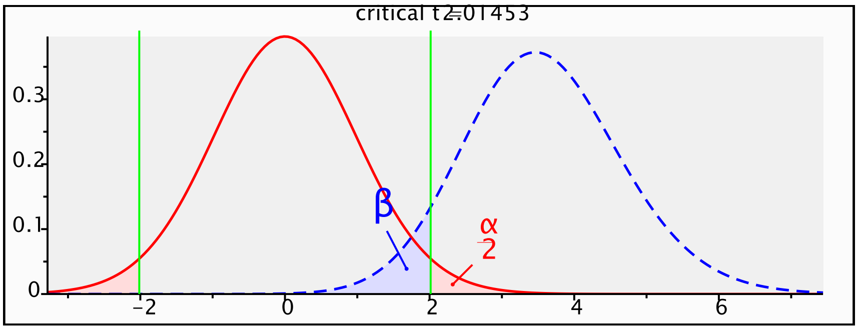

Ferulic acid

Mann-Whitney test: *p*-value = 0.0097

Cohen's D: 1.2181

Power post-hoc (1 – β err prob): 0.9540

False positive risk: 0.1289

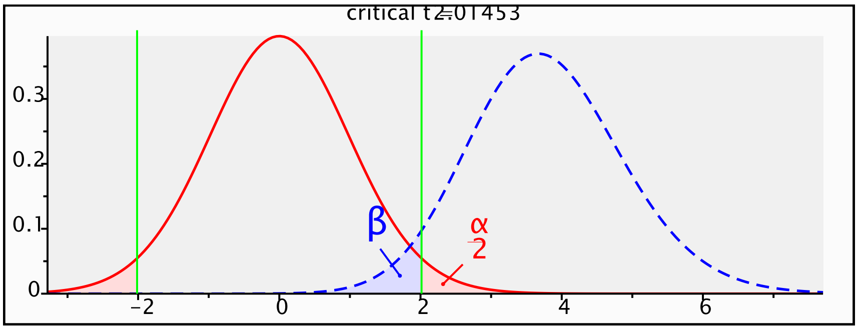

3-O-Methyl-a-methyldopa

Mann-Whitney test: *p*-value = 0,0164

Cohen's D: 1.3151

Power post-hoc (1 – β err prob): 0.9759

False positive risk: 0.3431

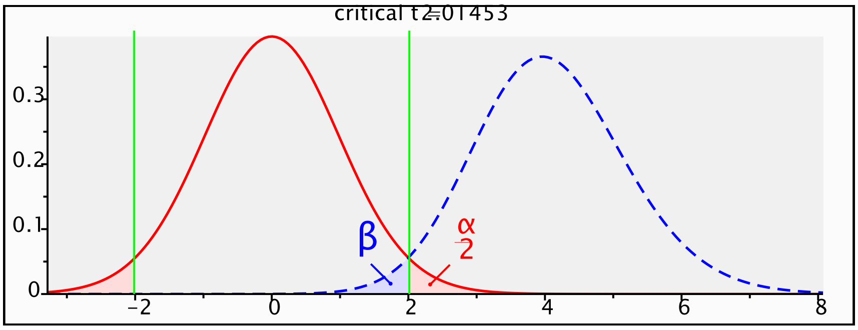

Hypoxanthine

Mann-Whitney test: *p*-value = 0,0001

Cohen’s D: 0.8191

Power post-hoc (1 – β err prob): 0.6886

False positive risk: 0.0025

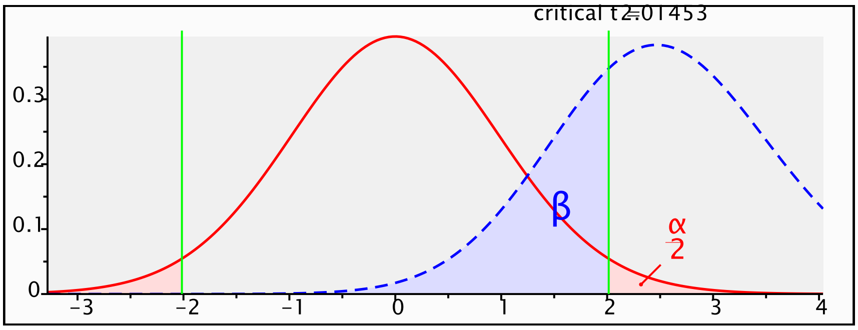

Chenodeoxycholic acid glycine conjugate [M-H]⁻

Mann-Whitney test: *p*-value = 0.0004

Cohen’s D: 0.8756

Power post-hoc (1 – β err prob): 0.7926

False positive risk: 0.0043

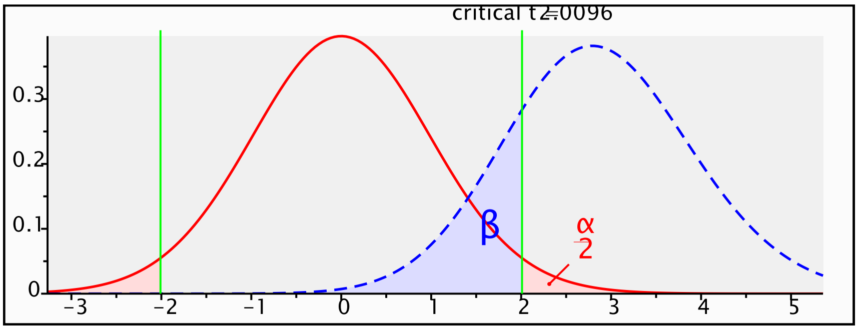

Caffeine

Mann-Whitney test: *p*-value = 0,0081

Cohen's D: 0.7885

Power post-hoc (1 – β err prob): 0.6555

False positive risk: 0.0539

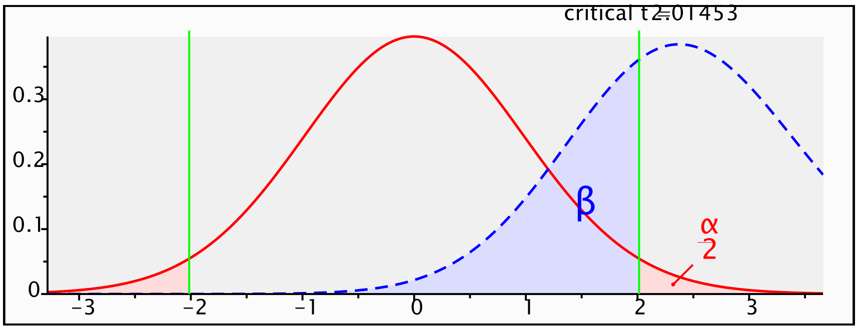

2-trans,4-cis-Decadienoylcarnitine

Mann-Whitney test: *p*-value = 0,0146

Cohen's D: 0.7000

Power post-hoc (1 – β err prob): 0.5539

False positive risk: 0.0895

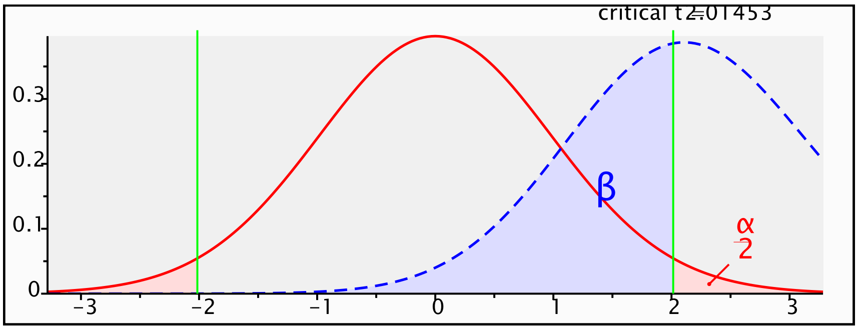

Pipecolic acid

Mann-Whitney test: *p*-value = 0,0072

Cohen's D: 0.6973

Power post-hoc (1 – β err prob): 0.5507

False positive risk: 0.054

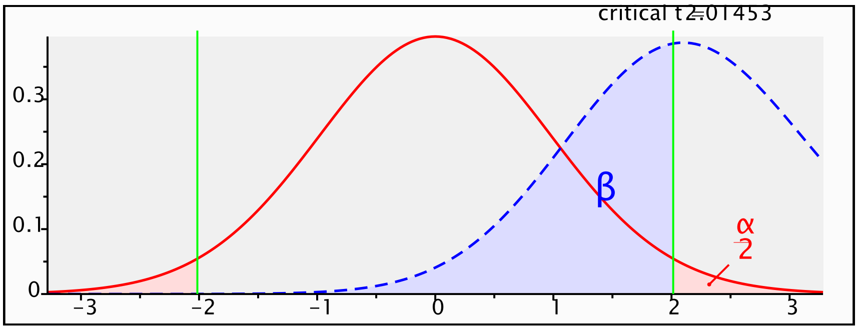

Paraxanthine

Mann-Whitney test: *p*-value = 0,0343

Cohen's D: 0.5980

Power post-hoc (1 – β err prob): 0.4327

False positive risk: 0.1726

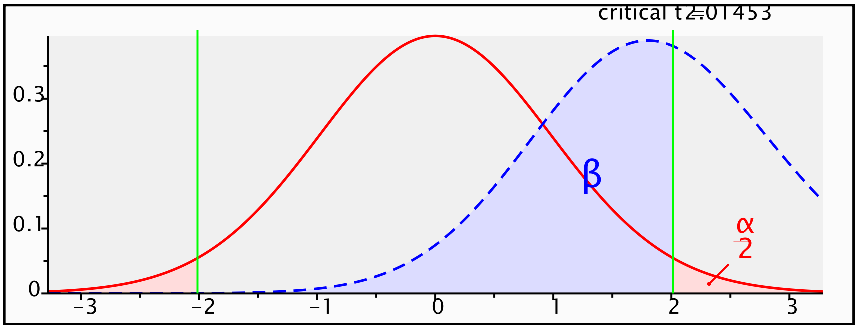

15(S)-HETE

Mann-Whitney test: *p*-value = 0,0204

Cohen’s D: 0.4924

Power post-hoc (1 – β err prob): 0.3139

False positive risk: 0.1472

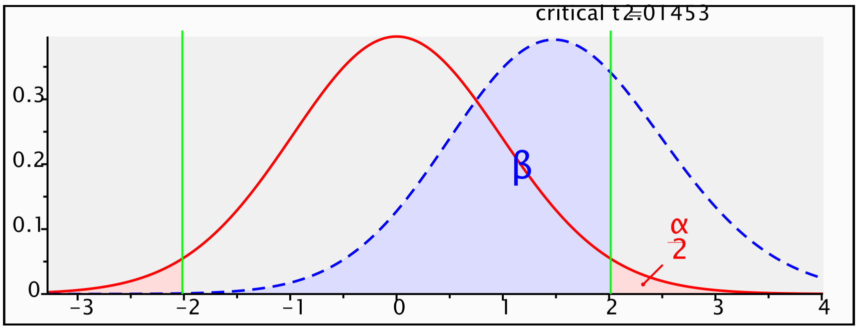

Taurodeoxycholic acid

Mann-Whitney test: *p*-value = 0.0017

Cohen’s D: 0.2741

Power post-hoc (1 – β err prob): 0.1300

False positive risk: 0.1594

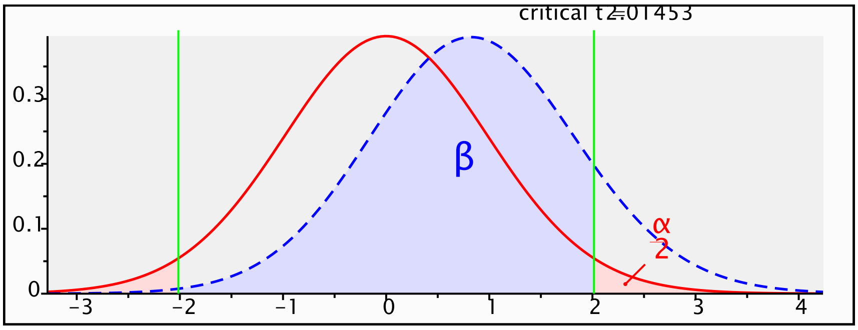

12-KETE

Mann-Whitney test: *p*-value = 0,0277

Cohen’s D: 0.4933

Power post-hoc (1 – β err prob): 0.3148

False positive risk: 0.1717

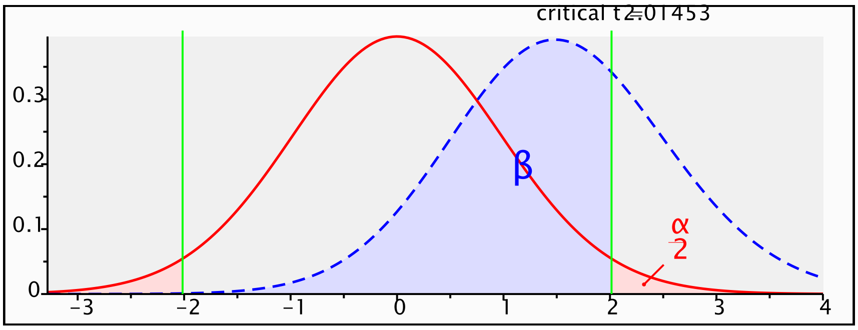

15(S)-HPETE

Mann-Whitney test: *p*-value = 0.0459

Cohen’s D: 0.6876

Power post-hoc (1 – β err prob): 0.5398

False positive risk: 0.2114

2. Supplementary Figures and Tables

Table S1. Parameters and values evaluated for the J48 method using the loop optimization node.

Method	Parameter	Range	Selection Value
J48	C_confidenceFactor	0-50	0.12
	M_minNumObj	1-200	2
	N_numfolds	1-200	3
	S_seed	1-5000	1
	Unpruned	True-False	False
	useLaplace	True-False	False
	useMDLcorrection	True-False	False
	subtreeRasing	True-False	True

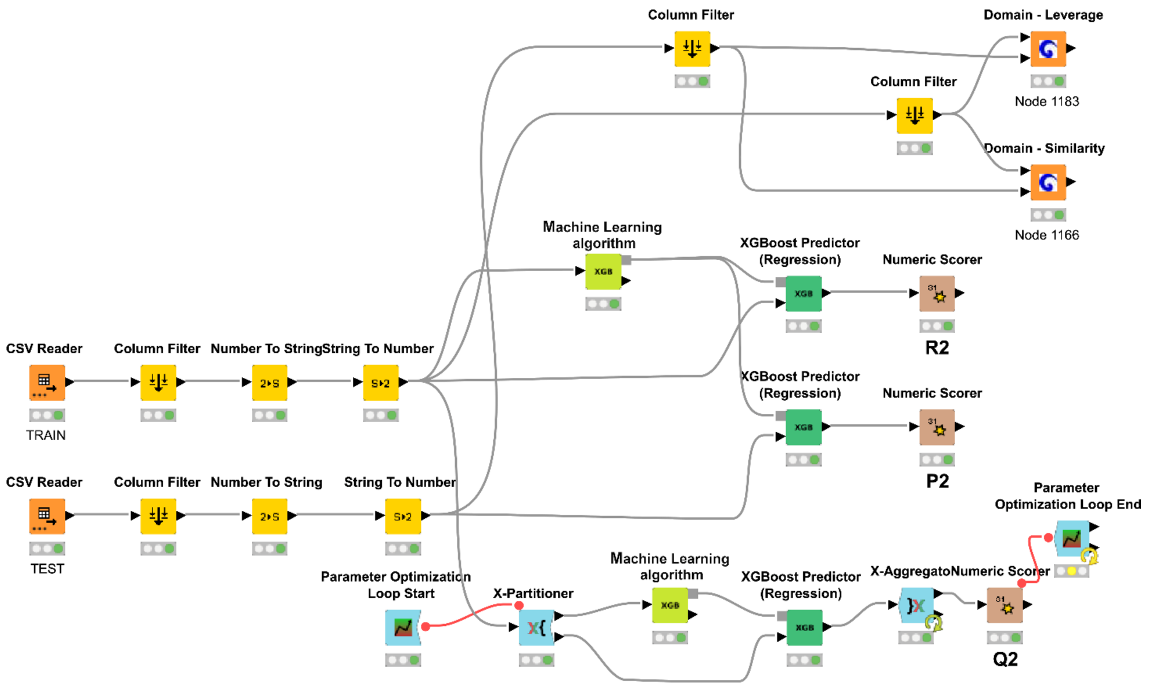

Figure S1. "X-Aggregator" and "Score" nodes combined for the model validation step.

3. Dataset for J48 Model - Complete, Test, and Training Sets.

Table S2. Complete J48 dataset model including8 VIPs

ID	Class	137.0463	181.0721	195.0887	414.3012	450.3217	457.2348	498.2884	195.0658
UNIFAL_T_ESIp_001	Control	712912	719188	1190021	119369	36491	328193	89337	4432
UNIFAL_T_ESIp_002	Patient	6693	1617	7141	1687	506	3434	159	2230
UNIFAL_T_ESIp_003	Control	1049839	911894	989302	192308	49653	72707	137661	14816
UNIFAL_T_ESIp_004	Patient	375123	2151686	1944536	62377	41832	14269	63991	179361
UNIFAL_T_ESIp_005	Patient	5876	906	7842	189	238	3812	37	1147
UNIFAL_T_ESIp_006	Patient	388023	4677	12564	220431	82498	15530	38820	87203
UNIFAL_T_ESIp_007	Patient	130037	731988	1800173	296389	73066	14646	18933	59965
UNIFAL_T_ESIp_008	Control	745441	261431	517392	141031	36304	109200	31681	23403
UNIFAL_T_ESIp_009	Patient	124208	1073005	1582217	213435	92875	11087	189913	306057
UNIFAL_T_ESIp_010	Control	796139	1046107	1760258	110975	26619	109314	63862	7478
UNIFAL_T_ESIp_011	Patient	30722	486500	2301598	137609	20350	3348	27707	4390
UNIFAL_T_ESIp_012	Patient	228011	1000561	1054854	45944	9863	7726	149552	149922
UNIFAL_T_ESIp_013	Control	863248	1835252	1589553	142448	28346	94524	128030	4874
UNIFAL_T_ESIp_014	Patient	1054993	16198	51966	98526	21789	105243	10778	19112
UNIFAL_T_ESIp_015	Patient	177128	866014	440706	42901	8212	3859	27086	10475
UNIFAL_T_ESIp_016	Patient	35	80	263	0	0	8589	45	62
UNIFAL_T_ESIp_017	Control	262298	235443	1595415	187036	67666	2676	79192	2434
UNIFAL_T_ESIp_018	Control	265264	231432	208792	124736	26338	1658	23413	4258
UNIFAL_T_ESIp_019	Patient	252148	452395	1179593	70039	14416	4467	9392	162347
UNIFAL_T_ESIp_020	Patient	356067	98236	655954	325598	49481	6533	32433	3532
UNIFAL_T_ESIp_021	Patient	148725	1656479	3445144	69645	55097	6876	119913	3928
UNIFAL_T_ESIp_022	Control	331963	534116	1015143	44547	20686	8607	34825	3027
UNIFAL_T_ESIp_023	Patient	29526	16988	36969	2594906	262370	1568	857074	6853
UNIFAL_T_ESIp_024	Patient	343909	9561	99803	29235	8492	11286	23851	3900

UNIFAL_T_ESIp_025	Control	191938	201195	270832	65914	12156	7122	17078	3235
UNIFAL_T_ESIp_026	Control	16943	1068465	2062392	86762	30385	192384	26434	4956
UNIFAL_T_ESIp_027	Control	213796	1839653	2908238	112213	38900	336816	36821	3099
UNIFAL_T_ESIp_028	Control	148247	1712288	3347982	223589	39286	30213	50050	4993
UNIFAL_T_ESIp_029	Control	543411	1399673	2696150	143870	26854	84350	36523	8152
UNIFAL_T_ESIp_030	Patient	123630	20224	46684	355877	19414	5633	127595	1625
UNIFAL_T_ESIp_031	Control	3548426	1601917	4158498	80722	22997	230367	12302	5271
UNIFAL_T_ESIp_032	Control	596950	276992	1111689	97811	19698	296107	25767	2333
UNIFAL_T_ESIp_033	Patient	278625	1147101	1997785	41024	9536	2973	2934	153812
UNIFAL_T_ESIp_034	Patient	106724	847066	849609	27401	7901	2579	26629	213739
UNIFAL_T_ESIp_035	Patient	107040	507930	495004	56287	8428	3836	12165	65781
UNIFAL_T_ESIp_036	Patient	406821	11702	14855	53492	46687	1840	53782	139288
UNIFAL_T_ESIp_037	Patient	78900	1041306	1054322	39580	47747	4003	140421	102397
UNIFAL_T_ESIp_038	Patient	185798	251840	244435	26461	8635	3478	11064	2593
UNIFAL_T_ESIp_039	Patient	85990	484527	489663	25418	6133	1269	11564	100664
UNIFAL_T_ESIp_040	Patient	162089	3837	6327	27776	9369	7283	8730	54629
UNIFAL_T_ESIp_041	Patient	118092	243919	96603	284278	70375	3805	86241	235957
UNIFAL_T_ESIp_042	Patient	120670	104508	87116	16026	23309	2387	84072	103711
UNIFAL_T_ESIp_043	Patient	150365	138016	760334	55350	9949	17228	11518	1993
UNIFAL_T_ESIp_044	Patient	134118	1445196	1408684	15639	2089	45655	20853	7085
UNIFAL_T_ESIp_045	Patient	86281	775384	508786	237161	42511	2083	87398	88967
UNIFAL_T_ESIp_046	Patient	203715	178054	309783	35578	11841	4915	9015	82954
UNIFAL_T_ESIp_047	Patient	248920	778397	1273708	52365	16405	1924	34163	2744
UNIFAL_T_ESIp_048	Patient	92113	1163955	2460550	19077	4411	1721	14868	5440
UNIFAL_T_ESIp_049	Patient	182326	1003584	1225762	43269	46828	7169	63396	5102
UNIFAL_T_ESIp_050	Patient	69386	793256	1575978	85231	13763	5051	9683	157539
UNIFAL_T_ESIp_051	Patient	197524	1457425	2775039	34340	10780	46585	19090	143965
UNIFAL_T_ESIp_052	Patient	148000	634960	823647	31188	10793	5226	15268	58723

UNIFAL_T_ESIp_053	Patient	129465	499908	930755	20222	5514	2282	5372	189598
UNIFAL_T_ESIp_054	Patient	85415	220761	437906	94893	21746	3389	20253	43856

File for download: https://doi.org/10.5281/zenodo.10960177

Table S3. J48 Test dataset model including8 VIPs.

ID	class	137.0463	181.0721	195.0887	414.3012	450.3217	457.2348	498.2884	195.0658
UNIFAL_T_ESIp_002	Patient	0.001876	0.000714	0.001654	0.00065	0.001929	0.006452	0.000142	0.007085
UNIFAL_T_ESIp_005	Patient	0.001646	0.000384	0.001823	7.28E-05	0.000907	0.007579	0	0.003546
UNIFAL_T_ESIp_006	Patient	0.109342	0.002137	0.002958	0.084948	0.314434	0.042501	0.045252	0.284779
UNIFAL_T_ESIp_011	Patient	0.008648	0.226073	0.55344	0.05303	0.077562	0.006196	0.032286	0.014144
UNIFAL_T_ESIp_015	Patient	0.049908	0.402459	0.105921	0.016533	0.031299	0.007719	0.031561	0.03403
UNIFAL_T_ESIp_018	Control	0.074746	0.107525	0.050148	0.04807	0.100385	0.001159	0.027275	0.013713
UNIFAL_T_ESIp_024	Patient	0.09691	0.004406	0.023938	0.011266	0.032367	0.029853	0.027786	0.012543
UNIFAL_T_ESIp_027	Control	0.060242	0.854977	0.699329	0.043244	0.148264	1	0.04292	0.009925
UNIFAL_T_ESIp_028	Control	0.041769	0.795781	0.805082	0.086165	0.149735	0.086259	0.058356	0.016115
UNIFAL_T_ESIp_029	Control	0.153133	0.650488	0.648325	0.055443	0.102352	0.247599	0.042572	0.026438
UNIFAL_T_ESIp_030	Patient	0.034831	0.009362	0.011164	0.137144	0.073995	0.013006	0.148836	0.005108
UNIFAL_T_ESIp_032	Control	0.168221	0.1287	0.267283	0.037693	0.075077	0.878679	0.030022	0.007422
UNIFAL_T_ESIp_034	Patient	0.030067	0.393653	0.204256	0.01056	0.030114	0.003904	0.031028	0.698302
UNIFAL_T_ESIp_036	Patient	0.11464	0.005402	0.003509	0.020614	0.177943	0.001702	0.06271	0.454994
UNIFAL_T_ESIp_040	Patient	0.04567	0.001746	0.001458	0.010704	0.035709	0.017923	0.010143	0.178326
UNIFAL_T_ESIp_042	Patient	0.033997	0.048535	0.020887	0.006176	0.08884	0.003332	0.098053	0.338728
UNIFAL_T_ESIp_053	Patient	0.036476	0.232305	0.223771	0.007793	0.021016	0.003019	0.006225	0.619409

File for download: https://doi.org/10.5281/zenodo.10960177

Table S4. J48 Train dataset model including 8 VIPs

ID	class	137.0463	181.0721	195.0887	414.3012	450.3217	457.2348	498.2884	195.0658
UNIFAL_T_ESIp_001	Control	0.200901	0.334219	0.286121	0.046001	0.139082	0.974302	0.104196	0.014281
UNIFAL_T_ESIp_003	Control	0.295854	0.423783	0.237851	0.07411	0.189248	0.2129	0.160581	0.048216
UNIFAL_T_ESIp_004	Patient	0.105707	1	0.467572	0.024038	0.159439	0.038743	0.074622	0.585954
UNIFAL_T_ESIp_007	Patient	0.036637	0.340168	0.432854	0.11422	0.278485	0.039866	0.022048	0.195765
UNIFAL_T_ESIp_008	Control	0.210069	0.121468	0.124363	0.054349	0.138369	0.321657	0.036923	0.076279
UNIFAL_T_ESIp_009	Patient	0.034994	0.498662	0.380439	0.082252	0.353985	0.02926	0.221549	1
UNIFAL_T_ESIp_010	Control	0.224356	0.486161	0.423255	0.042766	0.101456	0.321997	0.074472	0.024236
UNIFAL_T_ESIp_012	Patient	0.064248	0.464993	0.253615	0.017705	0.037592	0.019243	0.174456	0.489747
UNIFAL_T_ESIp_013	Control	0.243269	0.852931	0.382203	0.054895	0.108038	0.277919	0.149344	0.015726
UNIFAL_T_ESIp_014	Patient	0.297306	0.007491	0.012434	0.037969	0.083047	0.309864	0.012533	0.062256
UNIFAL_T_ESIp_016	Patient	0	0	0	0	0	0.021815	9.33E-06	0
UNIFAL_T_ESIp_017	Control	0.07391	0.109389	0.383613	0.072078	0.257903	0.004193	0.092359	0.007752
UNIFAL_T_ESIp_019	Patient	0.07105	0.210222	0.283613	0.026991	0.054945	0.009531	0.010916	0.530352
UNIFAL_T_ESIp_020	Patient	0.100336	0.04562	0.157685	0.125476	0.188592	0.015688	0.0378	0.01134
UNIFAL_T_ESIp_021	Patient	0.041903	0.769843	0.828448	0.026839	0.209997	0.01671	0.139873	0.012634
UNIFAL_T_ESIp_022	Control	0.093543	0.248203	0.244065	0.017167	0.078843	0.021869	0.040591	0.00969
UNIFAL_T_ESIp_023	Patient	0.008311	0.007858	0.008827	1	1	0.000891	1	0.022193
UNIFAL_T_ESIp_025	Control	0.054082	0.093472	0.065068	0.025401	0.046332	0.017443	0.019884	0.010369
UNIFAL_T_ESIp_026	Control	0.004765	0.496552	0.495914	0.033436	0.11581	0.569563	0.0308	0.015994
UNIFAL_T_ESIp_031	Control	1	0.744484	1	0.031108	0.087651	0.68276	0.014311	0.017023
UNIFAL_T_ESIp_033	Patient	0.078512	0.5331	0.480377	0.015809	0.036346	0.005078	0.00338	0.502459
UNIFAL_T_ESIp_035	Patient	0.030156	0.236033	0.118979	0.021691	0.032123	0.00765	0.014151	0.214771
UNIFAL_T_ESIp_037	Patient	0.022226	0.48393	0.253487	0.015253	0.181983	0.008148	0.163802	0.334434
UNIFAL_T_ESIp_038	Patient	0.052351	0.11701	0.05872	0.010197	0.032912	0.006583	0.012866	0.008271
UNIFAL_T_ESIp_039	Patient	0.024224	0.225156	0.117694	0.009795	0.023375	0	0.01345	0.32877
UNIFAL_T_ESIp_041	Patient	0.033271	0.113329	0.023168	0.109552	0.268228	0.007558	0.100584	0.770911

UNIFAL_T_ESIp_043	Patient	0.042366	0.064108	0.182787	0.02133	0.03792	0.047561	0.013396	0.006311
UNIFAL_T_ESIp_044	Patient	0.037787	0.671645	0.338706	0.006027	0.007962	0.13228	0.024288	0.022951
UNIFAL_T_ESIp_045	Patient	0.024306	0.360337	0.122293	0.091395	0.162027	0.002426	0.101934	0.290544
UNIFAL_T_ESIp_046	Patient	0.057401	0.082717	0.074435	0.013711	0.045131	0.010866	0.010476	0.270893
UNIFAL_T_ESIp_047	Patient	0.07014	0.361738	0.306247	0.02018	0.062526	0.001952	0.039819	0.008765
UNIFAL_T_ESIp_048	Patient	0.025949	0.540933	0.591666	0.007352	0.016812	0.001347	0.017305	0.017575
UNIFAL_T_ESIp_049	Patient	0.051373	0.466398	0.294716	0.016675	0.178481	0.017583	0.073928	0.016471
UNIFAL_T_ESIp_050	Patient	0.019544	0.368644	0.378938	0.032846	0.052456	0.011271	0.011255	0.514639
UNIFAL_T_ESIp_051	Patient	0.055656	0.677329	0.667297	0.013234	0.041087	0.135051	0.022231	0.470279
UNIFAL_T_ESIp_052	Patient	0.041699	0.295073	0.198013	0.012019	0.041137	0.011793	0.017772	0.191706
UNIFAL_T_ESIp_054	Patient	0.024062	0.102566	0.105247	0.036569	0.082883	0.006318	0.023588	0.14312

File for download: https://doi.org/10.5281/zenodo.10960177

4. Metabolomics Analysis with Mummichog

Table S5. Data from negative mode used on Mummichog analysis.

m/z	RT	p.value	t.stat
211.0609	1.841	0.000102	-4.1952
448.3655	5.799	0.000217	3.9659
210.0769	1.382	0.000396	-3.7779
919.6036	5.788	0.00049	3.7106
897.6224	5.795	0.001085	3.4531
919.6024	5.615	0.001296	3.3942
897.6207	5.61	0.0016	3.3237
913.6144	4.768	0.003269	3.0782
548.2319	5.799	0.003755	3.0294
150.0017	3.343	0.003921	-3.014
448.3065	5.784	0.00447	2.9673
241.0533	3.24	0.006439	-2.8348
448.3659	5.621	0.007192	2.7939
326.0878	1.427	0.007314	-2.7876
929.6105	4.767	0.008074	2.7508
311.2182	8.289	0.008276	-2.7415
548.2305	5.61	0.008434	2.7344
882.5318	7.011	0.00965	-2.6835
385.2228	4.013	0.010991	2.6338
403.1975	4.4	0.012051	-2.5983
448.3066	5.609	0.012661	2.5791
862.3953	2.581	0.012926	-2.5711
498.2885	5.32	0.015849	2.4909
543.3536	7.882	0.016624	-2.4719
523.725	2.528	0.01866	-2.4255
498.3519	5.116	0.024554	2.3131
391.1505	9.312	0.025324	2.3003
747.3687	2.311	0.025791	-2.2926
915.4778	8.436	0.026139	2.287
361.2013	4.445	0.026632	-2.2792
600.2602	4.526	0.026811	-2.2764
445.2438	4.939	0.026974	-2.2738

996.9757	2.77	0.027237	2.2698
345.1548	3.068	0.02973	2.2328
188.0091	2.392	0.036096	-2.1495
406.2623	5.64	0.0362	-2.1482
891.4788	8.451	0.039436	2.1108
443.2644	6.425	0.039702	-2.1078
410.0815	2.827	0.042053	-2.0825
869.3573	5.111	0.04244	-2.0784
993.6016	3.038	0.044241	2.0599
471.2413	5.565	0.044826	-2.054
287.186	5.031	0.044837	-2.0539
844.384	2.598	0.045594	-2.0464
621.3271	6.312	0.046587	-2.0368
311.1402	2.478	0.047799	-2.0252
653.2654	2.549	0.04883	-2.0156
289.1652	3.161	0.049853	-2.0062
897.6199	4.768	0.050302	2.0021
659.3337	6.097	0.051092	-1.9951
323.1678	7.968	0.051871	-1.9882
470.2566	3.931	0.052167	-1.9856
350.1453	2.54	0.052406	-1.9835
479.264	3.722	0.052923	-1.979
663.2662	3.322	0.053819	-1.9713
593.3342	3.492	0.054036	-1.9695
544.2581	3.357	0.054352	-1.9668

File for download: https://doi.org/10.5281/zenodo.10960177

319.2276	7.669	0.054758	1.9634
300.2179	6.327	0.056075	-1.9525
125.0966	6.443	0.056741	-1.947
315.2172	6.064	0.057	-1.9449
397.1708	2.163	0.059537	-1.9247
659.5138	9.482	0.059757	-1.923
719.5347	9.494	0.060117	-1.9202
688.783	2.517	0.061838	-1.9071
609.1976	2.299	0.061927	-1.9064
201.1126	3.529	0.06233	-1.9034
429.2485	6.065	0.065156	-1.8825
499.3091	5.308	0.065958	1.8768
259.1545	4.021	0.065982	-1.8766
934.4755	3.163	0.066743	1.8712
687.0593	2.414	0.066802	-1.8708
269.0232	1.909	0.0682	-1.8609
850.3283	2.461	0.070256	-1.8468
827.3586	2.563	0.07206	-1.8347
416.1778	1.393	0.072262	-1.8334
653.3013	7.853	0.073066	-1.8281
294.101	2.001	0.074547	-1.8184
852.575	7.869	0.076039	-1.8089
349.0377	3.378	0.076723	-1.8045
446.2901	5.221	0.078655	1.7925
815.3527	3.401	0.07974	-1.7858

301.1763	1.864	0.081215	-1.7769
257.2271	7.739	0.0814	-1.7758
672.2517	3.401	0.082004	-1.7722
629.1782	3.757	0.082655	-1.7683
743.4595	8.607	0.086064	-1.7485
315.192	1.963	0.086208	-1.7477
882.5338	2.974	0.088841	1.7328
657.3311	657.3311	0.089133	-1.7312
432.3105	6.829	0.089367	1.7299
687.3959	7.028	0.089544	-1.7289
661.2521	2.79	0.090697	-1.7225
438.213	1.76	0.090979	-1.721
531.0394	2.437	0.091054	-1.7206
604.3253	5.662	0.091063	1.7205
336.0114	3.275	0.092703	-1.7116
465.2491	4.658	0.09432	-1.703
785.5055	9.191	0.095462	-1.697
945.4662	5.099	0.096721	-1.6904
653.301	7.282	0.09742	-1.6868
283.1403	2.908	0.097433	-1.6867
674.3207	3.563	0.098015	-1.6837
331.129	3.493	0.098861	-1.6794
247.118	1.6	0.09903	-1.6785

Table S6. Data from positive mode used on Mummichog analysis.

m/z	RT	p.value	t.stat
457.2348	6.47	2.28E-05	4.6374
899.6373	5.639	3.33E-05	4.5276
414.3014	5.817	4.29E-05	4.4533
212.0927	1.381	6.23E-05	-4.3428
414.3016	5.637	0.000103	4.193
506.7572	2.242	0.00014	4.1001
450.3229	5.806	0.000145	4.0893
195.0658	1.381	0.000174	-4.0333
432.3118	5.645	0.000187	4.0116
472.3031	5.641	0.000196	3.9964
472.304	5.811	0.000233	3.9443
212.0928	0.681	0.000545	-3.6767
899.6388	5.82	0.000584	3.6543
466.3167	4.792	0.000773	3.564
294.1553	1.381	0.000794	-3.5554
621.6507	1.841	0.001131	3.4395
944.751	3.642	0.001196	-3.4209
430.2957	4.808	0.001294	3.3948
572.714	2.167	0.001316	3.3891
205.0679	0.508	0.001448	-3.3574
448.3428	6.649	0.001556	3.3332
412.2851	4.789	0.001892	3.267
339.269	5.811	0.002116	3.2289
572.5138	2.15	0.002472	3.1755
766.4127	2.851	0.002541	3.166
280.1403	0.658	0.002542	-3.1658
715.89	2.15	0.002568	3.1623
195.066	0.681	0.002721	-3.1423
944.5515	3.642	0.002781	-3.1347
137.0463	0.663	0.003172	3.0888
715.6399	2.15	0.003483	3.0559
294.1563	0.673	0.003631	-3.0412
448.3062	5.249	0.004074	3.0004
187.058	0.568	0.004096	2.9985
981.3912	4.374	0.00418	-2.9913
513.2146	6.525	0.004442	2.9695
184.2367	7.198	0.004676	2.9511
594.3016	3.356	0.004814	2.9406
284.0577	3.079	0.005023	-2.9253
243.1344	2.204	0.005336	2.9034
621.3168	1.841	0.005714	2.8785
240.0511	1.404	0.006026	2.8591
524.4366	8.402	0.006101	2.8546
481.169	2.391	0.006314	-2.842
352.0429	5.275	0.006342	2.8404
500.251	2.815	0.006573	2.8272
287.1969	0.59	0.007223	2.7923
991.7635	7.201	0.007305	2.7881
464.2837	5.127	0.007629	2.772
195.0887	2.024	0.007831	2.7622
624.9981	3.216	0.007877	2.76
944.6522	3.642	0.008708	-2.7224
624.6636	3.216	0.00927	2.6988
614.387	3.561	0.010684	2.6447
646.0322	6.246	0.011168	2.6277
666.3311	2.815	0.011827	2.6056
963.7129	7.704	0.012504	2.584
928.6375	3.671	0.013061	2.567
724.7957	3.736	0.013451	2.5556
276.145	0.673	0.013527	-2.5534
413.1701	3.056	0.013912	-2.5423
312.2175	3.356	0.01535	2.5036
181.0721	1.684	0.015433	2.5014
530.8458	3.14	0.015459	2.5008
596.3431	2.633	0.015545	2.4986
961.6975	7.706	0.017181	2.4587
809.5145	2.561	0.017267	2.4567
761.8157	3.906	0.017818	2.4441
184.2824	7.201	0.018632	2.4261
485.2147	4.457	0.018828	-2.4219
285.2222	7.774	0.020502	-2.3873
605.458	7.774	0.02055	-2.3864
834.6698	2.471	0.021108	2.3754
678.1107	3.011	0.021531	-2.3673
687.3605	3.184	0.021547	2.367
926.3516	3.056	0.021813	-2.362
638.3786	3.093	0.021942	2.3595
297.1681	4.548	0.022323	-2.3525
834.8365	2.469	0.022771	2.3443
887.5777	3.425	0.022779	2.3442

666.6651	2.815	0.023169	2.3372
747.3621	3.259	0.02337	2.3336
599.3085	3	0.025065	2.3045
666.2731	2.587	0.025118	-2.3037
560.3657	4.107	0.025679	2.2945
152.0169	3.379	0.027877	-2.26
583.3519	2.633	0.028739	2.2472
968.5794	3.368	0.028859	-2.2454
511.2555	2.778	0.029758	2.2324
775.4134	3.337	0.029779	2.2321
808.8804	3.155	0.030003	2.2289
215.128	3.149	0.030122	-2.2272
563.7946	2.001	0.030506	2.2218
604.3922	4.187	0.031483	2.2084
428.2859	3.804	0.032377	2.1964
199.1329	4.112	0.032605	-2.1934
377.2522	4.412	0.032983	2.1884
144.1109	0.596	0.033087	2.1871
697.3993	4.329	0.033493	2.1818
625.3322	3.216	0.033946	2.176
648.4185	4.262	0.03456	2.1683
384.2601	3.682	0.034797	2.1654
852.0787	2.966	0.035136	2.1612
516.3387	4.014	0.035323	2.1589
399.2652	4.461	0.035529	2.1563
269.1365	3.584	0.035538	-2.1562
963.646	8.261	0.036062	2.1499
472.3124	3.911	0.036412	2.1457

969.6512	9.455	0.036644	-2.1429
662.8314	2.988	0.036964	2.1391
653.3738	4.262	0.036979	2.1389
908.3367	3.764	0.037732	-2.1302
741.4266	4.403	0.037975	2.1273
650.75	3.529	0.038199	2.1248
899.7507	2.966	0.038476	2.1216
869.3299	3.068	0.03864	-2.1197
682.4049	3.184	0.038811	2.1178
518.3527	3.823	0.039039	2.1152
411.7485	3.472	0.039754	2.1073
609.3469	4.183	0.040298	2.1013
834.5023	2.46	0.040616	2.0978
257.1751	5.18	0.041437	-2.089
423.2023	2.506	0.041523	2.0881
550.3276	2.894	0.041694	2.0862
713.3735	4.333	0.041715	2.086
736.4702	4.407	0.0422	2.0809
692.4432	4.334	0.042703	2.0756
565.32	4.101	0.042861	2.074
547.3341	4.19	0.042889	2.0737
497.2368	2.483	0.043467	2.0678
263.2013	6.533	0.043796	2.0644
367.7208	3.34	0.044616	2.0561
680.372	3.011	0.045041	2.0519
819.4395	3.409	0.045118	2.0511
443.291	4.57	0.045843	2.044
118.0869	0.509	0.046538	2.0373

968.9092	4.018	0.04669	2.0358
732.3901	3.263	0.046829	2.0345
546.9278	5.897	0.047596	2.0271
503.3072	4.108	0.047848	2.0248
521.2934	4.012	0.047961	2.0237
467.2288	2.653	0.048281	2.0207
959.612	7.569	0.04889	2.015
482.2991	3.584	0.049473	2.0097
847.6024	2.988	0.05047	-2.0006
433.7617	3.516	0.050566	1.9998
690.8013	2.551	0.051397	-1.9924
651.0838	3.523	0.051422	1.9921
455.7749	3.584	0.051452	1.9919
824.7384	3.666	0.051847	-1.9884
776.3575	2.966	0.053165	1.9769
709.3808	2.587	0.053319	1.9756
192.033	0.658	0.054019	1.9696
688.3264	3.093	0.054181	-1.9683
176.144	2.127	0.056429	-1.9496
239.1653	7.167	0.056449	-1.9494
460.285	3.534	0.05771	1.9392
421.2784	4.522	0.058088	1.9362
780.5536	7.855	0.058215	-1.9352
690.4453	8.038	0.058828	-1.9303
683.8843	2.61	0.059046	1.9286
888.1992	3.368	0.059114	-1.9281
611.2137	2.31	0.059135	-1.9279
414.3012	4.781	0.059445	1.9255

798.5656	9.401	0.060489	-1.9174
863.4666	3.475	0.060546	1.9169
489.2784	2.77	0.061042	1.9131
987.6439	6.732	0.061097	1.9127
591.3599	4.268	0.061256	1.9115
562.3801	3.911	0.061321	1.911
555.2827	2.894	0.061638	1.9086
462.2746	2.655	0.06203	1.9056
445.1607	3.011	0.062073	-1.9053
585.2712	4.331	0.062452	1.9024
328.1124	2.988	0.064161	-1.8898
445.2797	6.454	0.064883	-1.8845
738.4805	4.218	0.065027	1.8835
453.252	2.77	0.065101	1.8829
770.4556	3.339	0.067028	1.8692
657.3441	2.529	0.068824	1.8566
274.275	4.951	0.07014	1.8476
327.2333	7.892	0.070706	-1.8438
789.4469	3.222	0.071922	1.8356
477.267	3.915	0.072179	1.8339

504.3114	3.65	0.074137	1.8211
175.1194	0.486	0.076117	-1.8084
542.3253	6.616	0.076189	1.8079
606.4079	4.001	0.076699	1.8047
657.3692	2.957	0.077607	1.799
433.2412	3.803	0.078225	1.7952
751.2808	3.808	0.078887	-1.7911
694.4603	4.149	0.079836	1.7853
805.4223	3.218	0.084275	1.7588
227.0893	1.752	0.084355	-1.7583
888.2835	3.355	0.085299	-1.7529
935.6122	6.43	0.085519	1.7516
663.2693	3.307	0.08627	-1.7473
740.2873	3.056	0.086347	-1.7469
358.3688	7.24	0.086732	1.7447
623.2141	3.114	0.087852	-1.7383
757.3994	4.401	0.088075	1.7371
688.3757	3.788	0.088635	-1.7339
670.9966	3.158	0.089198	1.7308
500.5011	2.815	0.089258	1.7305

780.4954	4.461	0.089299	1.7302
682.2868	2.105	0.089964	1.7266
138.0556	0.544	0.091205	1.7198
851.4867	3.958	0.09221	1.7143
432.7095	2.609	0.092461	-1.7129
497.2795	2.896	0.092616	1.7121
450.3217	4.79	0.092674	1.7118
247.1544	3.584	0.093289	-1.7085
563.5438	2.001	0.093829	1.7056
600.3372	2.966	0.094909	1.6999
729.8784	2.574	0.095336	-1.6976
749.3706	2.551	0.095596	-1.6963
716.3343	2.426	0.09615	1.6934
339.1998	7.164	0.096394	-1.6921
303.1922	1.886	0.096975	-1.6891
907.4917	3.538	0.097571	1.686
613.3413	2.862	0.09763	1.6857
232.147	1.551	0.098172	1.6829
981.5013	4.37	0.099054	-1.6784

File for download: https://doi.org/10.5281/zenodo.10960177

5. Normality test results

Table S7. Control group normality tests results.

Control Group		1-Lyso-2-arachidonoyl-phosphatidate	N-Docosahexaenoyl GABA	Vanillactic acid	3-O-Methyl-a-methyldopa	Chenodeoxycholic acid glycine conjugate	Caffeine	2-trans,4-cis-Decadienoylcarnitine	Pipecolic acid
**Descriptive parameters**	Mean	150982	917918	3859	5489	2093179	1766949	30180	278541
	Median	109200	927014	3011	3258	1800818	1595415	14009	169690
	RSD (%)	90.59%	67.03%	82.73%	107.80%	80.06%	62.68%	135.60%	84.12%
	Skewness	0.6655	0.7651	2.3390	3.0830	1.1670	0.5404	2.9180	1.6600
	Kurtosis	-0.6727	0.0555	7.0730	10.8900	0.8818	-0.2329	9.3750	2.5980
	Sample size (n)	15	15	15	15	15	15	15	15
**Normality tests (*P*-value)**	Anderson-Darling test	0.1015	0.2874	0.0045	<0,0001	0.0438	0.6757	<0,0001	0.0048
	D'Agostino & Pearson tes	0.3869	0.35	<0,0001	<0,0001	0.0716	0.593	<0,0001	0.0033
	Shapiro-Wilk test	0.0772	0.1822	0.001	<0,0001	0.0403	0.6266	<0,0001	0.0032
	Kolmogorov-Smirnov test	0.0492	>0,1000	0.0748	0.0034	0.0512	>0,1000	0.0015	0.0484
**Graphical parameters**		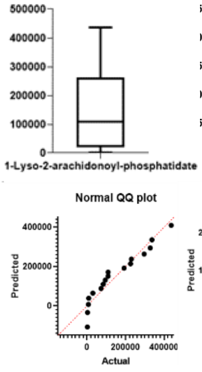	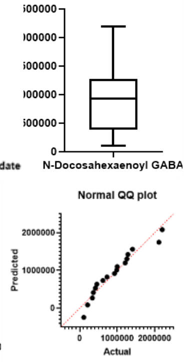	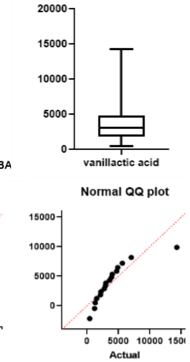	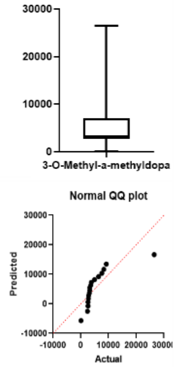	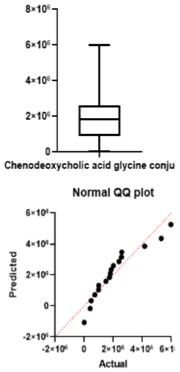	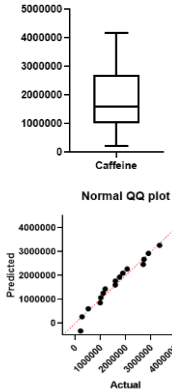	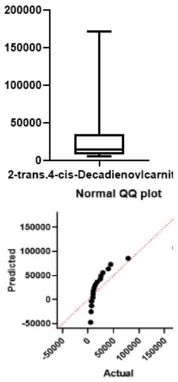	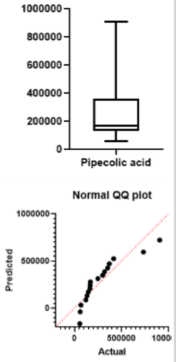

Control Group		Paraxanthine	15(S)-HETE	12-KETE	15(S)-HPETE	Taurodeoxycholic acid	Hypoxanthine	Ferulic acid
**Descriptive parameters**	Mean	1003835	1421643	22085	34725	733104	629612	6451
	Median	1046107	845098	2755	10433	489378	331963	4874
	RSD (%)	64.44%	102.30%	253.10%	155.10%	141.00%	128.60%	87,38%
	Skewness	0.1298	1.4650	3.0690	1.8130	3.4380	3.2270	2.387
	Kurtosis	-1.4730	2.1440	9.2900	2.4400	13.0100	11.8000	5.861
	Sample size (n)	15	15	15	15	15	15	15
**Normality tests (*P*-value)**	Anderson-Darling test	0.1518	0.0221	<0,0001	<0,0001	<0,0001	<0,0001	<0,0001
	D'Agostino & Pearson tes	0.0973	0.0097	<0,0001	0.0022	<0,0001	<0,0001	<0,0001
	Shapiro-Wilk test	0.0816	0.0116	<0,0001	<0,0001	<0,0001	<0,0001	0.0002
	Kolmogorov-Smirnov test	>0,1000	0.0287	<0,0001	<0,0001	0.0004	0.002	0.0003

Graphical parameters

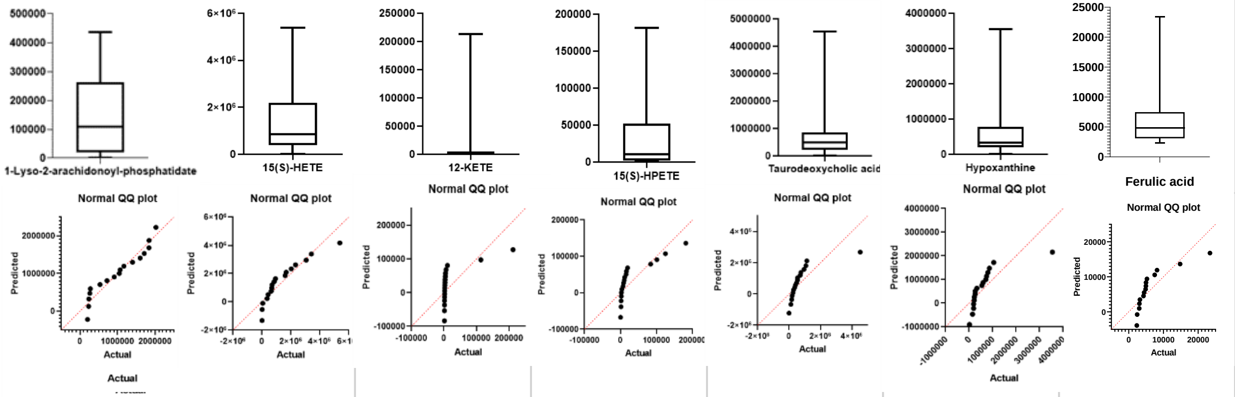

A distribution tends to be normal (Gaussian) as the mean approaches the median; the coefficient of variation is less than 30%; skewness values must be between -1 and +1; Kurtosis values (propensity for outliers) must be close to 0. The boxplot is symmetric and the points in the QQplot approach the line of identity.

Table S8. Patient group normality tests results.

Patients Group		1-Lyso-2-arachidonoyl-phosphatidate	N-Docosahexaenoyl GABA	Vanillactic acid	3-O-Methyl-a-methyldopa	Chenodeoxycholic acid glycine conjugate	Caffeine	2-trans,4-cis-Decadienoylcarnitine	Pipecolic acid
**Descriptive parameters**	Mean	10374	326299	100378	100030	1030653	884478	11647	155952
	Median	4467	133183	74755	84560	372674	655954	9274	93971
	RSD (%)	178.00%	156.80%	117.90%	100.90%	209.20%	100.00%	70.83%	98.09%
	Skewness	4.123	3.05	1.305	0.5414	3.643	1.062	0.8594	2.179
	Kurtosis	19.09	10.13	1.389	-1.001	13.82	0.6087	0.4252	5.229
	Sample size (n)	39	39	39	39	39	39	39	39
**Normality tests (*P*-value)**	Anderson-Darling test	<0,0001	<0,0001	<0,0001	<0,0001	<0,0001	0.0012	0.0607	<0,0001
	D'Agostino & Pearson tes	<0,0001	<0,0001	0.0022	0.0446	<0,0001	0.0198	0.0655	<0,0001
	Shapiro-Wilk test	<0,0001	<0,0001	<0,0001	0.0002	<0,0001	0.0007	0.0274	<0,0001
	Kolmogorov-Smirnov test	<0,0001	<0,0001	<0,0001	<0,0001	<0,0001	0.0145	>0,1000	<0,0001
**Graphical parameters**		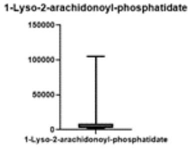 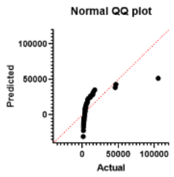	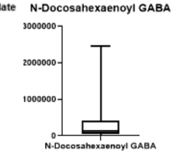 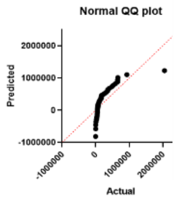	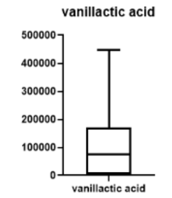 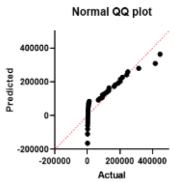	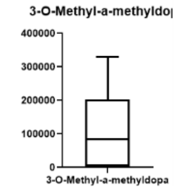 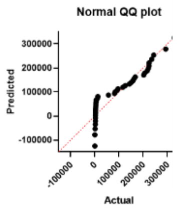	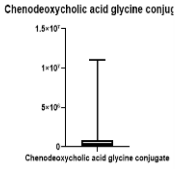 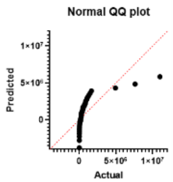	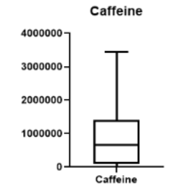 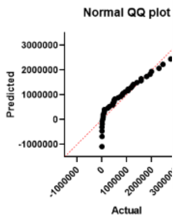	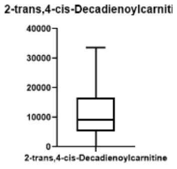 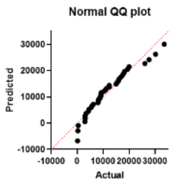	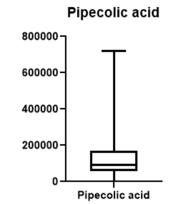 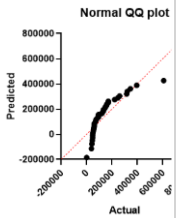

Patients Group		Paraxanthine	15(S)-HETE	12-KETE	15(S)-HPETE	Taurodeoxycholic acid	Hypoxanthine	Ferulic acid
**Descriptive parameters**	Mean	572301	796294	3932	6260	371559	183416	75965
	Median	486500	174473	1700	5485	131604	134118	58723
	RSD (%)	95.70%	156.60%	180.50%	86.66%	214.30%	97.20%	106,0%
	Skewness	0.8739	2.122	4.706	2.803	4.296	3.263	0.9525
	Kurtosis	0.3225	4.523	25.25	12.4	21.3	14.73	0.2329
	Sample size (n)	39	39	39	39	39	39	39
**Normality tests (*P* -value)**	Anderson-Darling test	0.0036	<0,0001	<0,0001	0.0004	<0,0001	<0,0001	<0,0001
	D'Agostino & Pearson tes	0.0665	<0,0001	<0,0001	<0,0001	<0,0001	<0,0001	0.0481
	Shapiro-Wilk test	0.0017	<0,0001	<0,0001	<0,0001	<0,0001	<0,0001	0.0002
	Kolmogorov-Smirnov test	0.0169	<0,0001	<0,0001	0.05	<0,0001	0.0005	0.0003
**Graphical parameters**		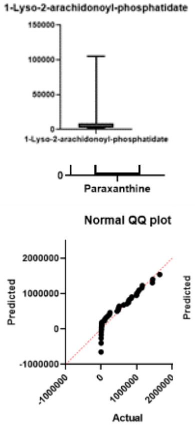	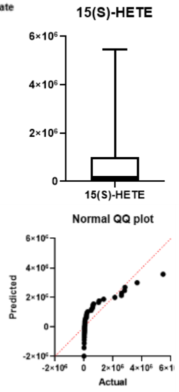	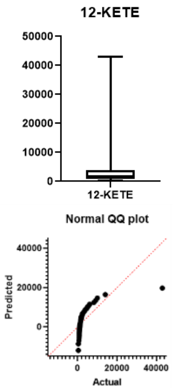	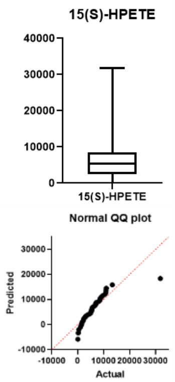	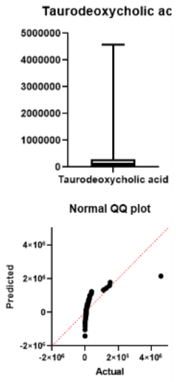	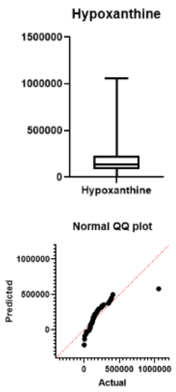	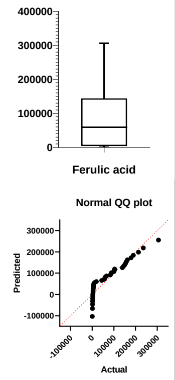

A distribution tends to be normal (Gaussian) as the mean approaches the median; the coefficient of variation is less than 30%; skewness values must be between -1 and +1; Kurtosis values (propensity for outliers) must be close to 0. The boxplot is symmetric and the points in the QQplot approach the line of identity.

6. VIP (Variable Importance in Projection) and Mass-to-Charge Ratios (*m/z*)

Table S9. VIP and *m/z*

m/z	Vip
457.2348	3.2878
899.6373	3.2316
414.3014	3.1929
212.0927	3.1344
211.0609	3.0543
414.3016	3.0531
506.7572	3.0017
450.3229	2.9956
195.0658	2.9642
432.3118	2.9519
472.3031	2.9433
448.3655	2.9259
472.304	2.9135
210.0769	2.8167
919.6036	2.7769
212.0928	2.7566
899.6388	2.7431
466.3167	2.6884
294.1553	2.6831
897.6224	2.62
621.6507	2.6116
944.751	2.6
430.2957	2.5836
919.6024	2.5833
572.714	2.5801
205.0679	2.5601
448.3428	2.5448
897.6207	2.5388
412.2851	2.5027
339.269	2.4782
572.5138	2.4438
766.4127	2.4376
280.1403	2.4375
715.89	2.4352
195.066	2.4222
944.5515	2.4172
137.0463	2.3872
913.6144	2.3802
715.6399	2.3655
294.1563	2.3558
548.2319	2.348
150.0017	2.3378
448.3062	2.3287
187.058	2.3275
981.3912	2.3227
513.2146	2.3082
448.3065	2.3067
184.2367	2.2958
594.3016	2.2888
284.0577	2.2785
243.1344	2.2638
621.3168	2.247
240.0511	2.2338
524.4366	2.2307
481.169	2.2222
352.0429	2.2211
241.0533	2.2173
500.251	2.2121
448.3659	2.1894
287.1969	2.1883
991.7635	2.1854
326.0878	2.1851
464.2837	2.1743
195.0887	2.1676
624.9981	2.1661
929.6105	2.1598
311.2182	2.1534
548.2305	2.1485
944.6522	2.1402
624.6636	2.1238
882.5318	2.1132
614.387	2.0862
385.2228	2.0786
646.0322	2.0743
666.3311	2.0588
403.1975	2.0537
963.7129	2.0436
448.3066	2.0402
862.3953	2.0345
928.6375	2.0317
724.7957	2.0236
276.145	2.022
413.1701	2.0142
312.2175	1.9867
181.0721	1.9852
530.8458	1.9847
596.3431	1.9832
498.2885	1.9777
543.3536	1.9642
961.6975	1.9547
809.5145	1.9533
761.8157	1.9443
184.2824	1.9314
523.725	1.931
485.2147	1.9284
285.2222	1.9035
605.458	1.9028
834.6698	1.8949
678.1107	1.889
687.3605	1.8888
926.3516	1.8852
638.3786	1.8834
297.1681	1.8783
834.8365	1.8724
887.5777	1.8723
666.6651	1.8672
747.3621	1.8646
498.3519	1.8497
599.3085	1.8435
666.2731	1.8428
391.1505	1.8404
560.3657	1.8361
747.3687	1.8348
915.4778	1.8307
361.2013	1.825
600.2602	1.8229
445.2438	1.8211
375.1805	1.8192
996.9757	1.8181
152.0169	1.811
583.3519	1.8015
968.5794	1.8002
345.1548	1.791
511.2555	1.7907

775.4134	1.7905
808.8804	1.7882
215.128	1.7869
563.7946	1.783
604.3922	1.7731
428.2859	1.7642
199.1329	1.762
377.2522	1.7584
144.1109	1.7574
697.3993	1.7535
625.3322	1.7492
648.4185	1.7435
384.2601	1.7413
852.0787	1.7382
516.3387	1.7365
399.2652	1.7346
269.1365	1.7345
963.646	1.7298
188.0091	1.7295
406.2623	1.7286
472.3124	1.7267
969.6512	1.7247
662.8314	1.7219
653.3738	1.7217
908.3367	1.7152
741.4266	1.7131
650.75	1.7112
899.7507	1.7088

869.3299	1.7075
682.4049	1.706
518.3527	1.7041
891.4788	1.7008
443.2644	1.6986
411.7485	1.6982
609.3469	1.6937
834.5023	1.6911
257.1751	1.6846
423.2023	1.6839
550.3276	1.6825
713.3735	1.6823
410.0815	1.6797
736.4702	1.6785
869.3573	1.6766
692.4432	1.6746
565.32	1.6734
547.3341	1.6732
497.2368	1.6687
263.2013	1.6662
993.6016	1.6628
367.7208	1.66
471.2413	1.6584
287.186	1.6584
680.372	1.6568
819.4395	1.6563
844.384	1.6527
443.291	1.6509

118.0869	1.6459
621.3271	1.6455
968.9092	1.6448
732.3901	1.6438
546.9278	1.6383
311.1402	1.6368
503.3072	1.6365
521.2934	1.6357
467.2288	1.6334
653.2654	1.6296
959.612	1.6292
482.2991	1.6251
289.1652	1.6225
897.6199	1.6195
847.6024	1.6183
433.7617	1.6177
659.3337	1.6141
690.8013	1.6121
651.0838	1.6119
455.7749	1.6117
824.7384	1.6091
323.1678	1.6089
470.2566	1.607
350.1453	1.6054
479.264	1.602
776.3575	1.6005
709.3808	1.5995
663.2662	1.5962

192.033	1.5949
593.3342	1.5948
688.3264	1.5939
544.2581	1.5928
319.2276	1.5902
300.2179	1.582
176.144	1.5798
239.1653	1.5796
125.0966	1.5778
315.2172	1.5762
460.285	1.5719
421.2784	1.5696
780.5536	1.5689
690.4453	1.5652
683.8843	1.5639
888.1992	1.5635
611.2137	1.5633
414.3012	1.5615
397.1708	1.5609
659.5138	1.5596
719.5347	1.5575
798.5656	1.5553
863.4666	1.555
489.2784	1.5521
987.6439	1.5518
591.3599	1.5509
562.3801	1.5505
555.2827	1.5487

688.783	1.5475
609.1976	1.547
462.2746	1.5464
445.1607	1.5462
201.1126	1.5447
585.2712	1.544
328.1124	1.5344
445.2797	1.5304
738.4805	1.5296
453.252	1.5292
429.2485	1.5289
499.3091	1.5244
259.1545	1.5243
934.4755	1.5202
687.0593	1.5199
770.4556	1.5186
269.0232	1.5124
657.3441	1.5091
274.275	1.5022
850.3283	1.5016
327.2333	1.4993
789.4469	1.493
827.3586	1.4923
477.267	1.4917
416.1778	1.4913
653.3013	1.4872
504.3114	1.4819
294.101	1.4798

852.575	1.4725
175.1194	1.4721
542.3253	1.4718
606.4079	1.4693
349.0377	1.4692
657.3692	1.4649
433.2412	1.462
446.2901	1.4599
751.2808	1.4588
815.3527	1.4548
694.4603	1.4544
301.1763	1.4479
257.2271	1.4471
672.2517	1.4443
629.1782	1.4413
805.4223	1.434
227.0893	1.4336
888.2835	1.4294
935.6122	1.4284
743.4595	1.426
315.192	1.4254
663.2693	1.4251
740.2873	1.4248
358.3688	1.4231
623.2141	1.4182
757.3994	1.4172
688.3757	1.4148
882.5338	1.4139

657.3311	1.4126
670.9966	1.4124
500.5011	1.4121
780.4954	1.4119
432.3105	1.4116
687.3959	1.4109
682.2868	1.4091
661.2521	1.406
438.213	1.4048
531.0394	1.4045
604.3253	1.4044
138.0556	1.4038
851.4867	1.3996
432.7095	1.3985
497.2795	1.3979
450.3217	1.3977
336.0114	1.3975
247.1544	1.3951
563.5438	1.3929
465.2491	1.3908
600.3372	1.3884
729.8784	1.3867
785.5055	1.3862
749.3706	1.3856
716.3343	1.3834
339.1998	1.3824
945.4662	1.3811
303.1922	1.38

653.301	1.3782
283.1403	1.3782
907.4917	1.3776
613.3413	1.3774
674.3207	1.3759
232.147	1.3752
331.129	1.3725
247.118	1.3718
981.5013	1.3717
360.1012	1.3664
445.2223	1.3647
887.5015	1.364
629.3588	1.3629
291.1494	1.3607
713.4479	1.3606
301.217	1.3596
699.286	1.3581
940.4495	1.3557
483.2928	1.3555
527.2144	1.3548
265.2536	1.3526
785.4528	1.3522
524.1452	1.351
362.1177	1.3504
704.7015	1.3487
783.1957	1.3477
858.5111	1.3441
538.1824	1.3436

924.3494	1.3387
680.2245	1.3384
888.0322	1.3361
443.3236	1.3351
774.567	1.3347
161.0217	1.3293
618.3279	1.326
194.045	1.324
740.4643	1.324
704.1204	1.3189
841.8323	1.3188
936.4923	1.3176
968.6705	1.3139
229.1442	1.3127
513.3433	1.3107
907.5803	1.3083
833.4741	1.3079
623.3279	1.3059
755.1809	1.3046
745.4204	1.3038
656.3106	1.3028
353.2125	1.3017
404.3153	1.2996
839.0912	1.2964
849.328	1.2957
528.3297	1.2945
653.1847	1.2918
406.2441	1.2901

918.3445	1.2894
714.2294	1.2889
701.3941	1.2874
160.0757	1.2868
858.6063	1.2851
620.9827	1.285
151.0256	1.283
544.2018	1.2822
802.0346	1.2814
401.2178	1.2811
963.645	1.2788
328.162	1.2772
798.5663	1.2758
884.603	1.2758
282.0544	1.2742
611.3796	1.2739
972.9528	1.2725
753.394	1.2725
445.2888	1.2718
427.1852	1.2671
660.1669	1.2663
978.3845	1.2646
498.2892	1.2628
981.6122	1.2618
600.0024	1.2602
334.1405	1.2599
660.3408	1.2595
551.2318	1.2589

223.0067	1.2574	464.3019	1.2151	691.1527	1.1721	961.6271	1.1365	652.3402	1.0948
599.3822	1.2566	240.9783	1.2124	330.1708	1.1707	587.0564	1.135	916.4614	1.0924
389.2178	1.2542	494.2572	1.2085	931.5032	1.1697	263.1035	1.1333	802.9721	1.0915
318.3012	1.2533	515.3037	1.2071	970.3737	1.1682	885.4715	1.1312	788.2266	1.0905
127.0411	1.2519	464.7961	1.2054	368.8976	1.1681	596.0087	1.1267	671.3508	1.0899
606.1873	1.2508	696.218	1.202	127.0018	1.1676	406.1248	1.1265	595.3504	1.0895
317.2111	1.2499	758.4144	1.201	213.0218	1.1671	818.555	1.1245	904.1451	1.0893
128.0348	1.2482	456.2587	1.201	342.03	1.166	618.5786	1.1237	725.4288	1.0877
327.6518	1.2462	211.1335	1.1998	648.6998	1.1655	820.2794	1.123	556.33	1.0875
885.5051	1.2438	150.0017	1.1949	362.3274	1.1651	313.1554	1.1192	554.1918	1.0866
397.1501	1.2419	330.1513	1.194	589.2809	1.1622	908.1938	1.1187	928.7816	1.0862
146.06	1.2418	797.4199	1.1939	381.7272	1.1613	603.2185	1.1181	918.2016	1.0848
645.3101	1.2417	498.288	1.1914	803.6777	1.1606	239.092	1.1169	803.5528	1.0842
649.0341	1.2362	495.0355	1.1901	507.2061	1.1601	386.3053	1.1169	649.2299	1.083
513.0853	1.2354	303.2329	1.1887	478.7576	1.1597	695.2646	1.1162	657.6781	1.0823
674.3204	1.2331	639.2078	1.1866	613.5042	1.1561	120.081	1.1153	297.1489	1.0807
755.4979	1.2306	403.7402	1.1858	841.446	1.1557	426.2025	1.1122	344.0273	1.0803
313.1628	1.2298	270.1246	1.1853	980.5987	1.1532	158.037	1.1099	756.1513	1.0783
302.3049	1.2287	665.3427	1.184	547.3335	1.1511	481.2437	1.1085	617.7358	1.0778
601.3268	1.2278	685.0342	1.1839	654.098	1.1445	651.3746	1.1056	846.8247	1.0774
716.9296	1.2274	426.2305	1.1815	553.311	1.1434	826.7748	1.1049	798.5058	1.0773
130.0869	1.2257	864.4111	1.1815	655.2816	1.1422	861.0279	1.1042	960.4888	1.0772
343.2272	1.225	963.5642	1.1787	735.3236	1.142	366.9005	1.1038	686.1969	1.0763
731.227	1.2238	621.3148	1.1782	483.2516	1.1418	418.1942	1.1028	930.9706	1.0761
824.522	1.2237	679.0131	1.1775	392.7502	1.1406	972.1077	1.101	439.1477	1.0749
586.8056	1.2228	169.0656	1.1752	830.7675	1.1404	583.256	1.1009	282.1254	1.0738
330.3377	1.2186	226.0174	1.1742	411.1727	1.1388	370.7373	1.0995	754.587	1.0734
847.0983	1.2159	619.7526	1.1741	758.0646	1.1376	882.0848	1.0977	370.8948	1.0732

352.1661	1.0731	868.5473	1.0635	619.7531	1.046	503.989	1.0312	414.7625	1.0099
786.3867	1.0723	425.7529	1.0629	274.1189	1.0455	371.2279	1.0311	688.4858	1.0099
343.0254	1.0714	419.1527	1.0621	480.2779	1.0455	698.3709	1.0246	600.323	1.0089
319.2272	1.0713	713.1346	1.0618	357.2794	1.0447	817.3822	1.0232	858.3243	1.0086
251.0593	1.0688	801.4317	1.0587	878.1073	1.0445	348.1014	1.0229	805.5598	1.0067
709.3677	1.0688	332.1411	1.057	972.5468	1.0438	339.1997	1.0221	346.333	1.0047
543.1898	1.0669	787.5219	1.057	591.3605	1.0429	921.5278	1.019	421.1688	1.0045
114.0667	1.0656	460.2398	1.0563	806.5346	1.0372	535.4004	1.0166	416.317	1.003
265.0927	1.0655	985.5132	1.056	696.4398	1.0365	378.1466	1.0154	463.2333	1.0028
900.001	1.0652	933.1045	1.0556	929.499	1.0363	595.6527	1.0141	489.3579	1.0021
379.2503	1.0651	335.2221	1.0522	725.3581	1.0334	756.4024	1.0139	508.1862	1.0003
742.7294	1.0645	938.9684	1.0504	854.54	1.0332	402.3942	1.0113		

File for download: https://doi.org/10.5281/zenodo.10960177

7. Volcano Plot Data

Table S10. Volcano test

	FC	log2(FC)	raw.pval	-LOG10(p)
414.3014	2.5876	1.3716	1.88E-05	4.725
899.6373	4.5378	2.182	5.36E-05	4.2712
450.3229	2.7961	1.4834	7.02E-05	4.1536
414.3016	3.8908	1.9601	7.67E-05	4.115
448.3655	3.1715	1.6652	8.38E-05	4.0766
432.3118	3.6565	1.8705	0.000129	3.8889
472.304	2.3686	1.2441	0.000129	3.8889
212.0927	0.027893	-5.1639	0.000196	3.7077
472.3031	3.4163	1.7724	0.000196	3.7077
211.0609	0.038243	-4.7087	0.000231	3.6369
919.6036	2.1672	1.1158	0.000271	3.5672
457.2348	5.6422	2.4963	0.000543	3.265
195.0658	0.066612	-3.9081	0.00063	3.2004
210.0769	0.039778	-4.6519	0.000844	3.0738
205.0679	0.28998	-1.786	0.000844	3.0738

466.3167	2.5511	1.3511	0.000844	3.0738
897.6207	2.7429	1.4557	0.000906	3.0427
944.751	0.38917	-1.3615	0.000906	3.0427
212.0928	0.045159	-4.4688	0.001045	2.9811
430.2957	2.3527	1.2343	0.001045	2.9811
919.6024	2.948	1.5597	0.001201	2.9203
621.6507	3.3033	1.7239	0.001288	2.8902
506.7572	8.2881	3.051	0.001477	2.8307
448.3065	2.4633	1.3006	0.001477	2.8307
448.3066	2.7808	1.4755	0.00169	2.772
339.269	2.0029	1.0021	0.001807	2.743
184.2824	3.5952	1.8461	0.00285	2.5452
481.169	0.1485	-2.7515	0.003036	2.5177
498.2885	2.3414	1.2274	0.003036	2.5177
184.2367	4.0174	2.0063	0.003233	2.4904
137.0463	4.1213	2.0431	0.003441	2.4633
412.2851	2.3654	1.2421	0.003441	2.4633
150.0017	0.056583	-4.1435	0.003662	2.4363
944.6522	0.42888	-1.2213	0.003894	2.4096
280.1403	0.40442	-1.3061	0.00414	2.383
897.6199	0.1087	-3.2016	0.004399	2.3567
294.1553	0.4284	-1.223	0.004672	2.3305
284.0577	0.064708	-3.9499	0.00496	2.3045
241.0533	0.33717	-1.5684	0.005264	2.2787
187.058	2.292	1.1966	0.005264	2.2787
548.2305	2.1066	1.0749	0.005264	2.2787
766.4127	2.0333	1.0238	0.005264	2.2787
929.6105	2.8297	1.5006	0.005585	2.253

448.3062	2.1004	1.0706	0.005922	2.2275
195.066	0.087764	-3.5102	0.006277	2.2023
287.1969	2.6701	1.4169	0.006651	2.1771
294.1563	0.44601	-1.1649	0.006651	2.1771
682.2868	0.24859	-2.0082	0.007044	2.1522
413.1701	0.27368	-1.8695	0.007044	2.1522
448.3659	2.0882	1.0623	0.007044	2.1522
991.7635	3.314	1.7286	0.007457	2.1274
599.2685	0.27919	-1.8407	0.007891	2.1028
311.2182	0.49953	-1.0013	0.007891	2.1028
646.0322	6.9176	2.7903	0.008827	2.0542
326.0878	0.25221	-1.9873	0.008827	2.0542
524.4366	3.0648	1.6158	0.009858	2.0062
464.3019	2.0752	1.0532	0.011601	1.9355
403.1975	0.026479	-5.239	0.012907	1.8892
195.0887	3.4527	1.7877	0.012907	1.8892
621.3168	3.3805	1.7572	0.013606	1.8663
666.2731	0.38836	-1.3645	0.013606	1.8663
276.145	0.49237	-1.0222	0.014337	1.8435
715.89	15.047	3.9114	0.015902	1.7985
605.458	0.17697	-2.4984	0.015902	1.7985
747.3687	0.48168	-1.0538	0.016738	1.7763
572.5138	16.795	4.07	0.019477	1.7105
543.3536	0.099912	-3.3232	0.020471	1.6889
215.128	0.10053	-3.3143	0.020471	1.6889
862.3953	0.18057	-2.4694	0.020471	1.6889
285.2222	0.25508	-1.971	0.020471	1.6889
572.714	13.878	3.7947	0.021508	1.6674

926.3516	0.2996	-1.7389	0.021508	1.6674
345.1548	2.5779	1.3662	0.02259	1.6461
963.7129	3.3153	1.7291	0.023717	1.6249
297.1681	0.16429	-2.6056	0.024892	1.6039
485.2147	0.23962	-2.0612	0.024892	1.6039
446.2901	2.2473	1.1682	0.024892	1.6039
523.725	0.35226	-1.5053	0.026115	1.5831
715.6399	15.451	3.9496	0.027389	1.5624
181.0721	3.0383	1.6033	0.027389	1.5624
899.7507	2.3251	1.2173	0.028715	1.5419
961.6975	3.2325	1.6927	0.030095	1.5215
996.9757	11.104	3.473	0.03153	1.5013
678.1107	0.10415	-3.2633	0.03153	1.5013
513.2146	8.6301	3.1094	0.033022	1.4812
199.1329	0.26258	-1.9292	0.034573	1.4613

600.2602	0.117	-3.0954	0.036184	1.4415
410.0815	0.027557	-5.1815	0.037858	1.4218
687.0593	0.039935	-4.6462	0.037858	1.4218
869.3299	0.28368	-1.8176	0.037858	1.4218
666.3311	3.2679	1.7084	0.037858	1.4218
653.2654	0.309	-1.6943	0.037858	1.4218
445.2438	0.094731	-3.4	0.039595	1.4024
849.328	0.33772	-1.5661	0.039595	1.4024
269.1365	0.13842	-2.8529	0.041398	1.383
611.2137	0.12485	-3.0017	0.043268	1.3638
118.0869	2.0226	1.0162	0.043268	1.3638
869.3573	0.036904	-4.7601	0.045208	1.3448
761.8157	5.7717	2.529	0.045208	1.3448
969.6512	0.45907	-1.1232	0.047219	1.3259

File for download: https://doi.org/10.5281/zenodo.10960177
